# ALKBH5 regulates somatic cell reprogramming in a phase-specific manner

**DOI:** 10.1242/jcs.259824

**Published:** 2022-06-10

**Authors:** Sherif Khodeer, Arne Klungland, John Arne Dahl

**Affiliations:** 1 Department of Microbiology, Oslo University Hospital, Rikshospitalet, Forskningsveien 1, 0373 Oslo, Norway; 2 Department of Biosciences, Faculty of Mathematics and Natural Sciences, University of Oslo, 0316 Oslo, Norway

**Keywords:** *Alkbh5*, Reprogramming, Induced pluripotent stem cells, iPSCs, SOX2, *Nanog*

## Abstract

Establishment of the pluripotency regulatory network in somatic cells by introducing four transcription factors [octamer binding transcription factor 4 (OCT4; also known as POU5F1), sex determining region Y (SRY)-box 2 (SOX2), Kruppel-like factor 4 (KLF4) and cellular myelocytomatosis (c-MYC)] provides a promising tool for cell-based therapies in regenerative medicine. Nevertheless, the mechanisms at play when generating induced pluripotent stem cells from somatic cells are only partly understood. Here, we show that the RNA-specific N6-methyladenosine (m^6^A) demethylase ALKBH5 regulates somatic cell reprogramming in a stage-specific manner through its catalytic activity. Knockdown or knockout of *Alkbh5* in the early reprogramming phase impairs reprogramming efficiency by reducing the proliferation rate through arresting the cells at G2/M phase and decreasing the upregulation of epithelial markers. On the other hand, ALKBH5 overexpression at the early reprogramming phase has no significant impact on reprogramming efficiency, whereas overexpression at the late phase enhances reprogramming by stabilizing *Nanog* transcripts, resulting in upregulated *Nanog* expression. Our study provides mechanistic insight into the crucial dynamic role of ALKBH5, mediated through its catalytic activity, in regulating somatic cell reprogramming at the post-transcriptional level.

This article has an associated First Person interview with the first author of the paper.

## Abstract (precis)

**Summary:** The biphasic regulatory role of ALKBH5 is mediated by its catalytic domain at both the early and late phase of somatic reprogramming.

## INTRODUCTION

The four transcription factors OCT4 (also known as POU5F1), SOX2, KLF4 and c-MYC (collectively OSKM) are sufficient to reprogram and induce pluripotency when ectopically expressed in mouse or human somatic cells to generate induced pluripotent stem cells (iPSCs) (Takahashi et al., 2007; Takahashi and Yamanaka, 2006). These reprogrammed iPSCs are highly similar to their pluripotent embryonic stem cell (ESC) counterparts in transcriptional profile and epigenetic landscape (Chin et al., 2009; Deng et al., 2009; Guenther et al., 2010), and show infinite self-renewal capability (Takahashi and Yamanaka, 2006) and the ability to differentiate into the three germ layers *in vivo* and *in vitro* (Carey et al., 2011). Therefore, iPSC technology provides an ideal tool for drug screening and patient-specific disease modeling, and holds great promise for therapeutic applications in the future (Onder and Daley, 2012).

The early phase of the reprogramming process is characterized by stochastic events (Buganim et al., 2012) in which mesenchymal genes are downregulated, while epithelial genes are upregulated in a process known as mesenchymal–epithelial transition (MET), together with clear morphological transformation accompanied by an increased proliferation rate to form cell clusters (Samavarchi-Tehrani et al., 2010; Li et al., 2010). However, most fibroblasts exposed to iPSC reprogramming conditions fail to achieve proper morphological changes and remain in a fibroblast like morphology. These trapped cells undergo senescence, apoptosis and cell cycle arrest, which in turn explain the low efficiency of the reprogramming process (Stadtfeld and Hochedlinger, 2010; Banito et al., 2009; Kim et al., 2018). In addition, several studies have demonstrated that cell cycle regulators, including p21 (also known as CDKN1A), p53 and p16 (also known as CDKN2A or INK4A), are barriers to the reprogramming process and that their depletion enhances the reprogramming process (Kawamura et al., 2009; Li et al., 2009; Hong et al., 2009; Utikal et al., 2009).

The late phase of the reprogramming process is considered deterministic, in which reactivation of endogenous *Sox2* expression is considered a rate-limiting step for acquiring ESC identity (Buganim et al., 2012). This phase is also characterized by removal of somatic epigenetic memory, telomere elongation, expression of endogenous pluripotency genes, and establishment of pluripotency specific epigenetic and transcriptional profiles (Li et al., 2010; Samavarchi-Tehrani et al., 2010).

The N6-methyladenosine (m^6^A) modification, methylation of the N6 position of the adenosine base, is the most abundant internal post-transcriptional modification in mammalian mRNA (Zhang et al., 2019). It was recently shown that m^6^A modification is reversible and that its presence is regulated through coordination of several modulators (Jia et al., 2011; Zheng et al., 2013). The positioning of m^6^A is mediated by methyl transferase-like 3 (METTL3), methyl transferase-like 14 (METTL14) and Wilms’ tumor 1-associating protein (WTAP) (Bokar et al., 1997; Schwartz et al., 2014; Liu et al., 2014; Ping et al., 2014). Removal of m^6^A is carried out by the demethylases fat mass and obesity-associated protein (FTO) and alkylated DNA repair protein AlkB homolog 5 (ALKBH5) (Gerken et al., 2007; Zheng et al., 2013). Furthermore, the m^6^A modification is recognized and bound by readers, including YTH domain-containing proteins 1–3 (YTHDF1–YTHDF3) and YTHDC1 and YTHDF2, which in turn facilitate downstream processing, such as mRNA splicing, stabilization, translation and degradation (Dominissini et al., 2012; Wang et al., 2014; Alarcón et al., 2015).

ALKBH5 is one of nine mammalian members of the AlkB family of Fe(II)- and α-ketoglutarate-dependent dioxygenases and can demethylate the m^6^A modification in RNA to adenosine (Zheng et al., 2013). We have previously shown that *Alkbh5* is highly expressed in meiotic cells of the testis and is mainly localized to the nucleus (Zheng et al., 2013). ALKBH5 has been shown to regulate various biological and pathophysiological processes including: meiosis, gametogenesis, autophagy, glioblastoma, breast cancer, lung cancer and infertility (Tang et al., 2018; Zheng et al., 2013; Song et al., 2019; Chao et al., 2020; Zhang et al., 2016; Zhang et al., 2017). In addition, the heterogeneity in *Alkbh5* expression in several cancer models has led to suggestions of a putative oncogenic or tumor suppressive role (Wang et al., 2020). Despite extensive studies on ALKBH5 in different biological systems, the functional and regulatory role of ALKBH5 in somatic cell reprogramming has not been addressed. In this study, we dissected the precise role of ALKBH5 in the reprogramming process, and our data revealed that ALKBH5 plays a biphasic role during somatic cell reprogramming. Depletion of *Alkbh5* in the very early phase of reprogramming impairs the reprogramming process through downregulation of cyclin B1 and B2, resulting in a reduction in the cell proliferation rate and arresting cells at G2/M phase accompanied by a decrease in the rate of MET. In the late phase, overexpression of ALKBH5 stabilizes *Nanog* transcripts, resulting in upregulated *Nanog* expression, which in turn enhances the reprogramming efficiency.

## RESULTS

### ALKBH5 depletion in the early phase impairs reprogramming efficiency

**Fig. 1.**
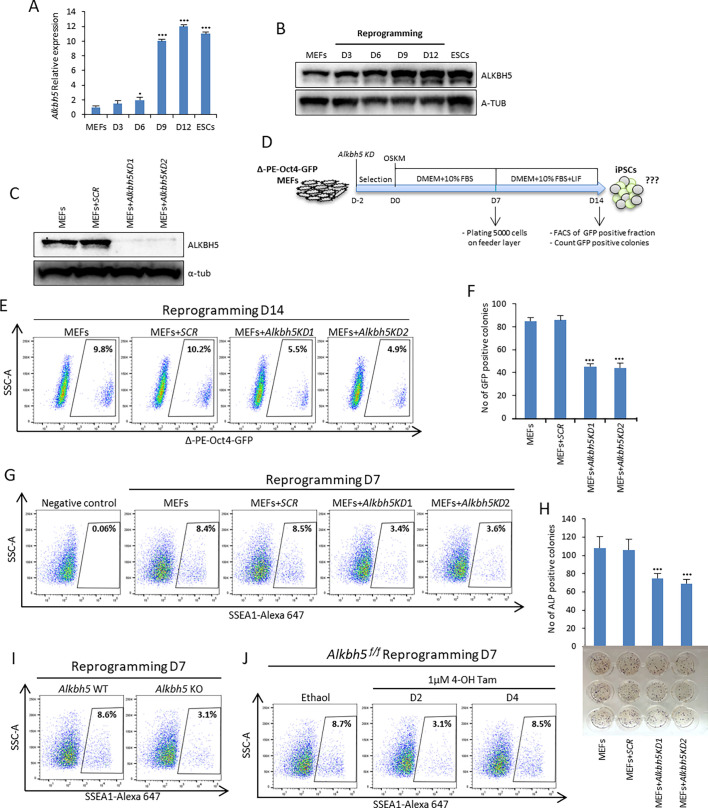
***Alkbh5* depletion impairs somatic cell reprogramming efficiency.** (A) Relative expression of *Alkbh5* during somatic cell reprogramming detected by qPCR. MEFs and mouse ESCs cultured in serum plus leukemia inhibitory factor (LIF), described as S/L, were used as negative and positive controls of pluripotency, respectively. Data are normalized to the housekeeping gene glyceraldehyde-3-phosphate dehydrogenase (*Gapdh*). (B) Immunoblot analysis of ALKBH5 protein levels during reprogramming. D, day. α-tubulin (A-TUB) was used as a loading control. (C) Immunoblot analysis of ALKBH5 protein levels in MEFs after lentiviral infection with either scrambled or two different shRNAs targeting *Alkbh5* (KD1 and KD2). Scrambled (*SCR*) shRNA used as a negative control. A-TUB was used as a loading control. (D) Experimental design showing the timing of *Alkbh5* knockdown, onset of reprogramming, counting of GFP-positive colonies and FACS analysis of the Δ-PE-Oct4-GFP-positive population. (E) Fraction of Δ-PE-Oct4-GFP-positive cells determined by FACS analysis after *Alkbh5* knockdown during the early phase of reprogramming. (F) Number of Δ-PE-Oct4-GFP-positive colonies on day 14 of reprogramming. (G) Fraction of SSEA1-positive cells determined by FACS analysis after *Alkbh5* knockdown during the early phase of reprogramming. Negative control is unreprogrammed MEFs. (H) Reprogramming efficiency was measured by counting the number of ALP-positive colonies. (I) Fraction of SSEA1-positive cells determined by FACS for reprogrammed wild-type (WT) and knockout (KO) *Alkbh5* MEFs assessed at day 7 of reprogramming. (J) Fraction of SSEA1-positive cells determined by FACS analysis of reprogrammed homozygous floxed *Alkbh5* (*Alkbh5^f/f^*) treated with ethanol as a control or 1 µM 4-OH Tam for depletion of *Alkbh5* at either day 2 or day 4. Images in B, C, E, G, I and J are representative of three repeats. Quantitative data are shown as the mean±s.d.; *n*=3. *P<0.05, ***P<0.001 (paired Student's *t*-test).

To explore the role of ALKBH5 in reprogramming, we first examined the expression of *Alkbh5* during the reprogramming process in mouse embryonic fibroblasts (MEFs), and we found that the expression of ALKBH5 was gradually upregulated during reprogramming at both the mRNA and protein levels (Fig. 1A,B). Then, we used two different short hairpin RNAs (shRNAs) to knockdown *Alkbh5* expression (Fig. 1C). As expected by knocking down *Alkbh5*, we found that the total m^6^A level at mRNA was highly increased compared to the controls (Fig. S1A).

Next, we established a reprogramming system in which *Alkbh5* was knocked down 2 days before induction of retroviral reprogramming factors (OSKM) in mouse embryonic fibroblasts (MEFs). We used Δ-PE-Oct4-GFP transgenic reporter MEFs (OG2 MEFs) in which GFP expression is encoded by the distal enhancer regulatory region of Oct4 as a stringent marker for establishment of a naive pluripotency network (Rais et al., 2013; Mor et al., 2018; Velychko et al., 2019). Then, we assessed the reprogramming efficiency by counting the number of GFP-positive colonies and by flow cytometry of the GFP-positive fraction on day 14 (Fig. 1D). Interestingly, *Alkbh5* knockdown significantly reduced the reprogramming efficiency by decreasing both the percentage of the Δ-PE-Oct4-GFP-positive fraction and the number of GFP-positive colonies on day 14 (Fig. 1E,F). Furthermore, we established another reprogramming system using non-transgenic MEFs and assessed the reprogramming efficiency by measuring the percentage of stage-specific embryonic antigen 1 (SSEA1)-positive cells (an early reprogramming marker) on day 7 and the number of alkaline phosphatase (ALP)-positive colonies on day 14 (Fig. S1B). In agreement with our previous data, *Alkbh5* knockdown significantly reduced the reprogramming efficiency by decreasing the percentage of SSEA1-positive cells on day 7 and the number of ALP-positive colonies on day 14 compared to controls (Fig. 1G,H). In addition, we used flow cytometry to assess the percentage of the Δ-PE-Oct4-GFP-positive population gated on the SSEA1-positive fraction. Our data revealed that knockdown of *Alkbh5* reduced the percentage of the Δ-PE-Oct4-GFP-positive population compared to the control (Fig. S1C). To substantiate these data, we derived *Alkbh5*-knockout (KO) MEFs and found that the reprogramming efficiency of *Alkbh5* KO MEFs was greatly reduced compared to that of wild-type (WT) MEFs on both day 7 and day 14 as revealed by the decreased percentage of SSEA1-positive cells (Fig. 1I; Fig. S1D,E) (Zheng et al., 2013). Taken together, these data suggest that *Alkbh5* depletion at the early phase of reprogramming impairs somatic cell reprogramming.

To further characterize the time-specific role of ALKBH5, we took advantage of a doxycycline (Dox)-inducible short hairpin RNA (shRNA) expression system to suppress the expression of *Alkbh5* at specific time points during reprogramming (Fig. S1F). We found that *Alkbh5* knockdown at the very early stage of reprogramming, earlier than day 3, had the largest impact on reducing the reprogramming efficiency, as shown by the decreased fraction of SSEA1-positive cells on days 7 and 14 of reprogramming (Fig. S1G). On the other hand, we did not see any significant change in reprogramming efficiency when *Alkbh5* was knocked down specifically at a later time than day 3 of the reprogramming process (Fig. S1G,H). To confirm our data, we used Δ-PE-Oct4-GFP reporter MEFs coupled with a Dox-inducible *Alkbh5* knockdown system and treated the cells with Dox for 2 days, for seven different time intervals (day 0–2, 2–4, 4–6, 6–8, 8–10, 10–12 and 12–14) throughout the whole reprogramming process until day 14. Then, we assessed the reprogramming efficiency on day 14 using flow cytometry. Consistent with our previous results, we found that only knockdown of *Alkbh5* in the very early stage, day 0–2 and day 2–4, impaired the reprogramming efficiency (Fig. 1G,H; Fig. S1I).

Furthermore, we derived homozygous floxed *Alkbh5* (*Alkbh5^f/f^*) MEFs, and we used a 4-hydroxytamoxifen (4-OH Tam)-inducible Cre recombinase system in which Cre was flanked by mutated ligand-binding domains of the murine estrogen receptor (Mer-Cre-Mer) to deplete *Alkbh5* at specific time points during reprogramming (Fig. S1J–L) (Zheng et al., 2013). Consistent with our time-specific knockdown data, depletion of *Alkbh5* only at the very early stage (day 2) of reprogramming impaired reprogramming, as measured by a decreased percentage of SSEA1-positive cells in the population (Fig. 1J). Time-specific depletion of *Alkbh5* at day 8 or 10 of reprogramming had no significant impact on the reprogramming efficiency (Fig. S1M). We further confirmed our data by treating homozygous floxed *Alkbh5* MEFs with 4-OH Tam to deplete *Alkbh5* at different time points of reprogramming, and we found that only *Alkbh5* depletion on day 2 or day 4 had a major impact on reducing the reprogramming efficiency, as measured by ALP staining at day 14 (Fig. S1N). In conclusion, only *Alkbh5* depletion at the very early stage of reprogramming negatively affects the reprogramming process.

### ALKBH5 regulates reprogramming through its m6A demethylase activity

**Fig. 2.**
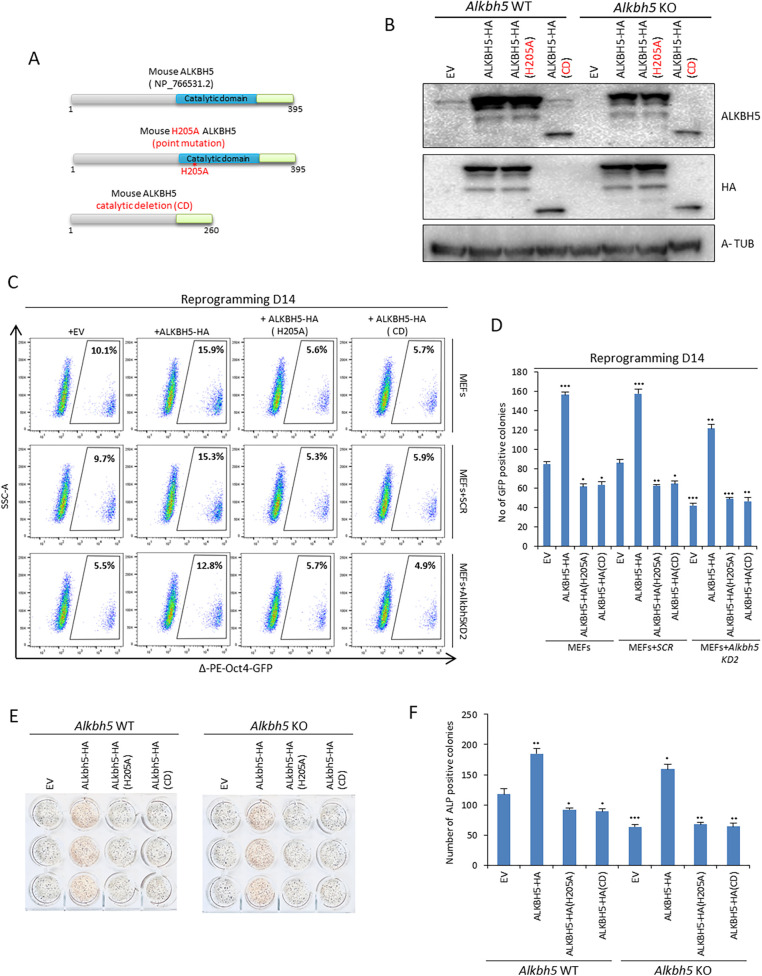
**ALKBH5 regulates reprogramming through its catalytic activity.** (A) Schematic representation of mouse ALKBH5 protein. Upper panel represents the wild-type (WT) ALKBH5. The middle panel represents catalytically inactive ALKBH5 with a point mutation in the catalytic domain, in which the histidine at position 205 is converted into alanine. The lower panel represents the catalytically deleted (CD) form of ALKBH5. (B) Immunoblot analysis of ALKBH5 protein levels after overexpression of the HA-tagged forms of ALKBH5–HA, ALKBH5–HA (H205A) and ALKBH5–HA (CD) in either WT or KO *Alkbh5* MEFs on day 3 of reprogramming. α-tubulin (A-TUB) was used as a loading control. (C) Fraction of Δ-PE-Oct4-GFP-positive cells determined by FACS analysis after overexpression of ALKBH5–HA, ALKBH5–HA (H205A) and ALKBH5–HA (CD) in uninfected MEFs or infection with either scrambled (SCR) shRNA or shRNA targeting the *Alkbh5* 3′UTR (KD2) on day 14 of reprogramming. (D) Number of Δ-PE-Oct4-GFP-positive colonies on day 14 of reprogramming. EV, empty vector. (E) Representative image of ALP staining on day 14. (F) Reprogramming efficiency was measured by counting the number of ALP-positive colonies presented in Fig. 2E. Images in B and C are representative of three repeats. Quantitative data are shown as the mean±s.d.; *n*=3. *P<0.05, **P<0.01, ***P<0.001 (paired Student's *t*-test).

Next, we asked whether the regulatory effect of ALKBH5 on somatic reprogramming is due to its m6A demethylase activity. We constructed two different mutants of ALKBH5 tagged with a C-terminal hemagglutinin (HA) tag. The first one has a point mutation in the catalytic domain in which histidine at position 205 is replaced with alanine to create catalytically inactive mouse ALKBH5 (H205A) (Zheng et al., 2013). In the second one, we completely deleted the catalytic domain to create catalytically dead ALKBH5 (CD) (Fig. 2A; Fig. S2A). Then, we overexpressed WT ALKBH5–HA, ALKBH5–HA (H205A) or ALKBH5–HA (CD) in both WT and KO *Alkbh5* MEFs, and we confirmed the overexpression by immunoblotting (Fig. 2B; Fig. S2B). Next, we assessed the effect of overexpression on reprogramming using Δ-PE-Oct4-GFP by counting the number of GFP-positive colonies and by flow cytometry on day 14. We found that overexpression of WT ALKBH5–HA enhances the reprogramming process as assessed on day 14, and it was able to rescue the reduction in reprogramming efficiency elicited by shRNA construct number 2, which targets the 3′ untranslated region (3′UTR) of *Alkbh5* (Fig. S2C)*.* Interestingly, overexpression of either ALKBH5–HA (H205A) or ALKBH5–HA (CD) was not able to rescue the knockdown of *Alkbh5,* but decreased the reprogramming efficiency in WT MEFs (Fig. 2C,D). To further validate our data, we overexpressed either WT ALKBH5–HA, ALKBH5–HA (H205), or ALKBH5–HA (CD) on day 1 of reprogramming in both WT and KO *Alkbh5* MEFs. Our data revealed that overexpression of ALKBH5–HA enhanced reprogramming efficiency by increasing the number of ALP-positive colonies in both WT and KO *Alkbh5* MEFs (Fig. 2E,F). However, overexpression of ALKBH5–HA (H205A) or ALKBH5–HA (CD) decreased the number of ALP-positive colonies in WT MEFs and was not able to increase the number of ALP-positive colonies in KO *Alkbh5* MEFs (Fig. 2E,F). In addition, our analysis of the SSEA1-positive fraction on day 7 and day 14 by flow cytometry revealed that overexpression of WT ALKBH5–HA in the early phase of reprogramming until day 7 did not have any impact on reprogramming efficiency. However, overexpression of WT ALKBH5–HA in the late stage of reprogramming, after day 7, increased reprogramming efficiency. In addition, overexpression of ALKBH5–HA (H205A) or ALKBH5–HA (CD) in WT or KO *Alkbh5* MEFs reduced the reprogramming efficiency as assessed by a decrease in the fraction of the SSEA1-positive cells (Fig. S2D). It is worth mentioning that *Alkbh5* KO MEFs exhibited a reduced proliferation rate compared to WT MEFs, and overexpression of WT ALKBH5–HA in *Alkbh5* KO MEFs restored the proliferation rate. Overexpression of either ALKBH5–HA (H205A) or ALKBH5–HA (CD) was unable to restore the proliferation rate in *Alkbh5* KO MEFs, and surprisingly decreased the proliferation rate in *Alkbh5* WT MEFs (Fig. S2E). Taken together, our data reveal that ALKBH5 regulates somatic cell reprogramming through its m6A demethylase activity. Overexpression of either the ALKBH5–HA (H205A) or ALKBH5–HA (CD) had a negative effect on the reprogramming process and was not able to rescue the phenotype of *Alkbh5* KO MEFs.

### Effect of *Alkbh5* removal during the early phase of reprogramming on cell cycle regulators and MET

**Fig. 3.**
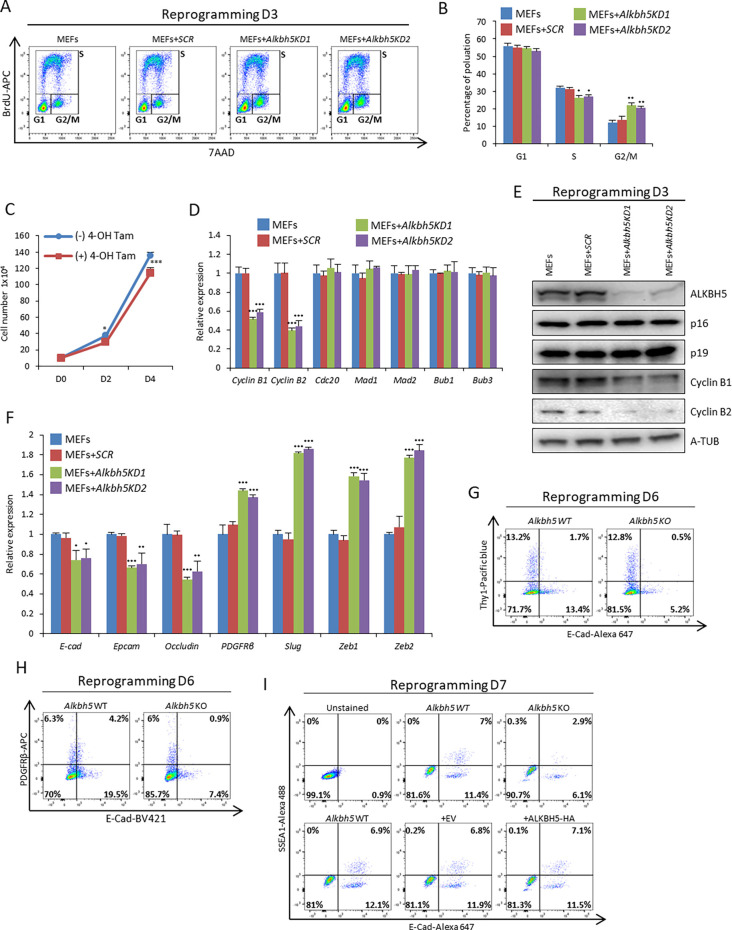
***Alkbh5* depletion induces G2/M cell cycle arrest and impairs the MET process.** (A) Cell proliferation of reprogrammed MEFs was assessed by FACS measured by BrdU incorporation on day 3 of reprogramming using either scrambled shRNA or two different shRNAs targeting *Alkbh5*. (B) Quantification of the mean percentage of each of the populations G1, S, and G2/M from FACS data shown in A. (C) Cell proliferation was assessed by counting *Alkbh5^f/f^* cells with or without the addition of 1 µM 4-OH Tam for *Alkbh5* depletion. (D) Expression of mitotic checkpoint complex (MCC) factors as assessed by qPCR on day 3 of reprogramming using either scrambled shRNA (*SCR*) or two different shRNAs targeting *Alkbh5* (KD1 and KD2). The data were normalized to the level of the housekeeping gene *Gapdh*. (E) Immunoblot analysis of the protein levels of several cell cycle regulators on day 3 of reprogramming using either scrambled shRNA or two different shRNAs targeting *Alkbh5*. α-tubulin (A-TUB) was used as a loading control. (F) Expression of mesenchymal and epithelial genes as assessed by qPCR on day 6 of reprogramming after infection either with scrambled shRNA or two different shRNAs targeting *Alkbh5*. The data were normalized to the housekeeping gene *Gapdh*. (G) Estimation of E-cadherin (E-cad) and Thy-1-positive populations by FACS in WT and *Alkbh5* KO cells on day 6 of reprogramming. (H) Estimation of E-cad- and PDGFRβ-positive populations by FACS in WT and KO *Alkbh5* MEFs on day 6 of reprogramming. (I) Estimation of E-cad- and SSEA1-positive populations by FACS in WT and *Alkbh5* KO MEFs or MEFs infected with either empty vector (EV) or ALKBH5–HA on day 7 of reprogramming. Images in A, E, G–I are representative of three repeats. Quantitative data are shown as the mean±s.d.; *n*=3. *P<0.05, **P<0.01, ***P<0.001 (paired Student's *t*-test).

To investigate the mechanism involved in reduced reprogramming efficiency resulting from loss of *Alkbh5*, we focused on two important events – cell proliferation and MET – which have both been reported to be critical to the early phase of reprogramming (Li et al., 2010; Samavarchi-Tehrani et al., 2010). First, we explored the impact of *Alkbh5* removal on proliferation and apoptosis during the early phase of reprogramming. Our data revealed that *Alkbh5* knockdown during the early phase of reprogramming increased the percentage of cells at G2/M phase (Fig. 3A,B). Additionally, *Alkbh5* depletion resulted in reduced cell proliferation during reprogramming, which is consistent with our data on *Alkbh5* KO MEFs (Fig. 3C; Fig. S2E). However, we did not observe any significant changes in the percentage of Annexin-positive cells as compared to the control, indicating that the reduction in cell number is mainly due to G2/M cell cycle arrest and not due to cell apoptosis (Fig. S3A–C). Overexpression of ALKBH5–HA during reprogramming did not have any impact on the cell cycle phases or cell proliferation rate (Fig. S3D–F). Next, we assessed the expression of factors of the mitotic checkpoint complex (MCC) and found that cyclin B1 and B2 were markedly downregulated at both the RNA and protein levels after knocking down ALKBH5 during the early phase of reprogramming (Fig. 3D,E). Other MCC factors, such as *Cdc20*, *Mad1* (*Mad1l1*), *Mad2* (*Mad1l2*), *Bub1* and *Bub3*, or G1 phase cell cycle regulators, such as *p16* and *p19* (*Cdkn2d*), were not significantly affected (Fig. 3D,E; Fig. S3G). To validate our *Alkbh5* knockdown data, we used *Alkbh5^f/f^* MEFs and induced *Alkbh5* removal by 4-OH Tam 8 h after reprogramming induction. In agreement with our knockdown data, we found a reduction in cyclin B1 and B2 levels, showing that this phenotype presents with the loss of *Alkbh5* both in MEFs and in the early reprogramming process (Fig. S3H). It is also noteworthy that depletion of *Alkbh5* in MEFs decreased the proliferation rate and induced cell cycle arrest at G2/M phase accompanied by a reduction in the protein levels of both cyclin B1 and B2 (Fig. S3I–L). This is consistent with what we observed during reprogramming (Fig. 3A–E; Fig. S2E). The phenotype of *Alkbh5* KO MEFs urged us to eliminate the possibility that nonretroviral infected MEFs have an impact on the readout of reprogramming efficiency. We assessed the infection efficiency using retroviral pMXs-DsRed as a control. Our infection efficiency was higher than 90%, as estimated by flow cytometry (Fig. S3M–O) (Okita et al., 2010). Then, we assessed the reprogramming efficiency using double gating of both DsRed and SSEA1 on day 7 and day 14 in WT and KO *Alkbh5* MEFs with or without ALKBH5–HA overexpression. Our data revealed that the SSEA1-positive population emerges from the DsRed-positive population, and the percentage of SSEA1-positive cells is decreased in KO *Alkbh5* MEFs on both day 7 and day 14, but increased on day 14 in the case of ALKBH5–HA overexpression compared to WT MEFs (Fig. S3P). To further support these data, we applied the piggyBac (PB) transposon reprogramming system in which the polycistronic reprogramming cassette (OSKM) is under the tetracycline regulatory (Tet-ON) promoter and separated from the mCherry fluorescent protein by an internal ribosome entry site (IRES) for simultaneous tracking of the reprogrammed population (Fig. S3O,Q) (Kim et al., 2015). Our data obtained with the PB transposon reprogramming system were very similar to what was seen with the retroviral reprogramming results, which not only substantiated our findings but also indicated that the regulatory role of ALKBH5 in reprogramming is relevant to both reprogramming methodologies (Fig. S3R). Thereafter, we assessed the MET process at day 6 of reprogramming in which *Alkbh5* was knocked down 2 days before reprogramming induction (Fig. S3S). Our quantitative (q)PCR and western blot data revealed that *Alkbh5* depletion impairs the MET process by decreasing the rate of downregulation of mesenchymal markers such as platelet-derived growth factor receptor β (*Pdgfrb*), Snail family zinc finger 2 (*Slug*, also known as *Snai2*), zinc finger E-box binding homeobox 1 (*Zeb1*) and zinc finger binding homeobox 2 (*Zeb2*), and through reduced upregulation of epithelial markers such as E-cadherin (*E-cad*; also known as *Cdh1*), epithelial cell adhesion molecule (*Epcam*) and occludin (*Ocln*) (Fig. 3F). To precisely estimate the change in MET, we used flow cytometry to assess the percentage of cells-positive for both E-Cad and thymocyte differentiation antigen-1 (Thy-1), as a mesenchymal marker, on day 6 of reprogramming in both *Alkbh5* WT and KO MEFs. Reprogramming of *Alkbh5* KO MEFs resulted in a reduced fraction of E-Cad-positive cells as compared to WT MEFs, whereas we did not see any significant difference in the fraction of Thy1-positive cells (Fig. 3G). Furthermore, to strengthen our findings, we repeated the same experiment using another mesenchymal marker, PDGFRβ, together with E-Cad, which clearly indicated a reduction in the fraction of the E-Cad-positive cells on day 6 of reprogramming of *Alkbh5* KO MEFs as compared to WT MEFs, whereas we did not see any significant difference in the fraction of PDGFRβ-positive cells (Fig. 3H). In addition, we used *Alkbh5^f/f^* MEFs and found that depletion of *Alkbh5* by using 4-OH Tam resulted in a reduction in the fraction of E-cad-positive cells on day 6 of reprogramming, whereas we did not see any significant difference in the fraction of Thy1 or PDGFRβ-positive cells (Fig. S3T–V). It is noteworthy that the discrepancy between PDGFRβ RNA and protein levels might due to post-transcriptional regulation. Moreover, to gain more insight into MET in the context of reprogramming, we used flow cytometry to assess both SSEA1 and E-Cad-positive populations on day 7 and 14 of reprogramming. Our data revealed that the SSEA1-positive population emerged from the E-Cad-positive population, and *Alkbh5* KO MEFs showed a reduction in the percentage of both the single-positive E-Cad population and double-positive SSEA1 and E-Cad population compared to WT MEFs on both day 7 and day 14 (Fig. 3I; Fig. S3W). Although ALKBH5–HA overexpression did not have any effect on MET or reprogramming efficiency on day 7, its overexpression in the late stage of reprogramming increased both the fraction of single-positive E-Cad cells and the fraction of double-positive SSEA1 and E-Cad cells as compared to WT MEFs (Fig. 3I; Fig. S3W). In addition, we used Δ-PE-Oct4-GFP MEFs and flow cytometry to assess the E-Cad-positive and Δ-PE-Oct4-GFP-positive populations on day 14 of reprogramming. Our data revealed that the Δ-PE-Oct4-GFP-positive population emerges from the E-Cad-positive population and that knockdown of *Alkbh5* decreases both the fraction of E-Cad single-positive cells and the fraction of double-positive Δ-PE-Oct4-GFP and E-Cad cells as compared to the WT control (Fig. S3X). The role of ALKBH5 in MET is further supported by our observations of morphological changes during reprogramming after *Alkbh5* depletion (Fig. S3Y). In summary, *Alkbh5* is required for proper cell proliferation and for proper upregulation of epithelial markers during the early phase of reprogramming.

### Cyclin B1 and B2 are downstream targets of ALKBH5

**Fig. 4.**
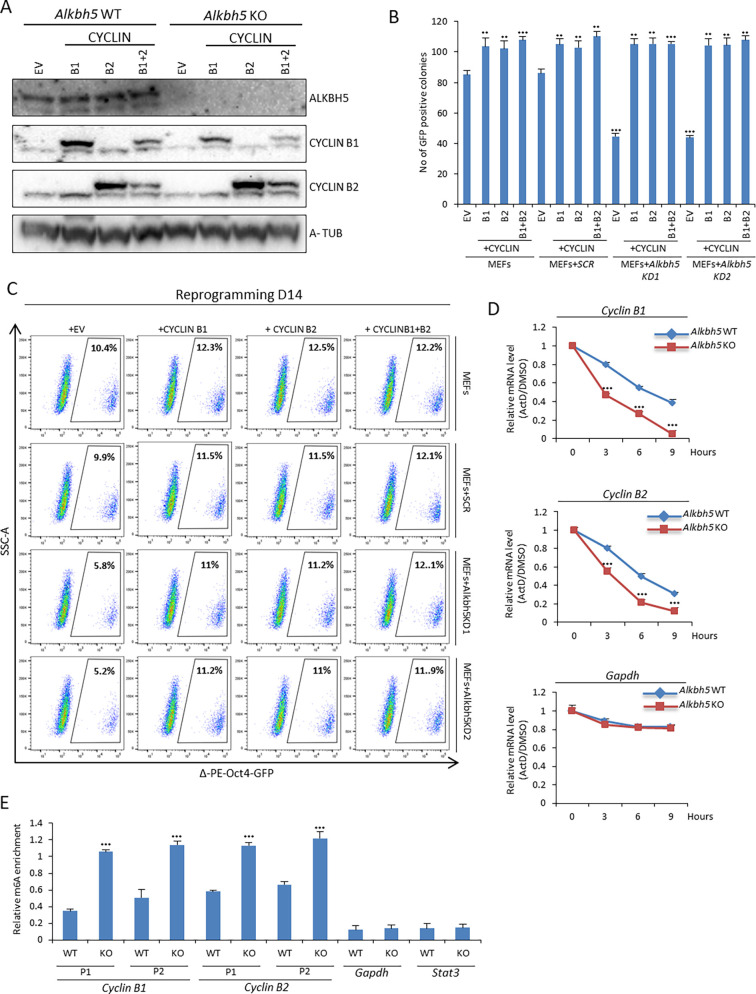
**Overexpression of cyclin B1 and/or B2 enhances the reprogramming efficiency in WT and *Alkbh5* KO MEFs.** (A) Immunoblot analysis of cyclin B1 and B2 overexpression in WT and *Alkbh5* KO MEFs on day 3 of reprogramming. α-tubulin (A-TUB) was used as a loading control. (B) Number of Δ-PE-Oct4-GFP-positive colonies on day 14 of reprogramming. (C) Fraction of Δ-PE-Oct4-GFP-positive cells at day 14 of reprogramming determined by FACS analysis after overexpression of cyclin B1 and/or B2 in uninfected MEFs or infection with either scrambled shRNA (*SCR*) or two different shRNAs targeting *Alkbh5* (KD1 and KD2). (D) Stability of cyclin B1 and B2 mRNA on day 3 of reprogramming. Both WT and *Alkbh5* KO MEFs were treated with either DMSO or 5 µM actinomycin D (ActD) at different time points from 0 to 9 h. *Gapdh* was used as a negative control, and the data from cells treated with ActD were normalized to that of DMSO-treated cells. (E) m^6^A-IP qPCR data of cyclinB1 and B2 mRNA on day 3 of reprogramming in WT and *Alkbh5* KO MEFs using two different primers sets (P1 and P2). Both primers were designed to span the m^6^A-rich region of cyclin B1 and B2 mRNA transcripts. *Gapdh* and *Stat3* were used as negative controls. Images in A and C are representative of three repeats. Quantitative data are shown as the mean±s.d.; *n*=3. ***P<0.001 (paired Student's *t*-test).

Our data revealed that depletion of *Alkbh5* induced cell cycle arrest at G2/M phase, accompanied by a reduction in the expression of both cyclin B1 and B2 (Fig. 3A–E). Next, we asked whether overexpression of cyclin B1 and/or B2 can compensate for *Alkbh5* depletion during reprogramming. We overexpressed cyclin B1 and B2 individually or together in both *Alkbh5* WT and KO MEFs (Fig. 4A; Fig. S4A). We found that overexpression of either cyclin B1 or B2, or both together, enhanced cell proliferation in both *Alkbh5* WT and KO MEFs during reprogramming (Fig. S4B). Then, we used Δ-PE-Oct4-GFP to assess the role of cyclin B1 and/or B2 in reprogramming. Our data revealed that overexpression of either cyclin B1 or B2, or both together, enhanced the reprogramming efficiency as measured by an increased number of GFP-positive colonies and an increased fraction of Δ-PE-Oct4-GFP-positive cells on day 14 of reprogramming (Fig. 4B,C). Furthermore, we applied ALP staining and flow cytometry to assess the fraction of SSEA1-positive cells on day 7 and day 14 of reprogramming in *Alkbh5* WT and KO MEFs overexpressing either cyclin B1 or B2, or both. Our data showed that overexpression of either cyclin B1 or B2, or both, enhanced the reprogramming efficiency in *Alkbh5* WT MEFs as assessed by an increased fraction of SSEA1-positive cells on day 7 and day 14 accompanied with increasing in the cell number (Fig. S4B,C). Overexpression of either cyclin B1 or B2, or both, restored the fraction of SSEA1-positive cells in *Alkbh5* KO MEFs on day 7, while the reprogramming efficiency increased on day 14 compared to control MEFs (Fig. S4B,C). Our ALP staining data showed that overexpression of either cyclin B1 or B2, or both, enhanced the reprogramming efficiency in both *Alkbh5* WT and KO MEFs (Fig. S4D,E). To further explore the mechanism of cyclin B1 and B2 mRNA (*Ccnb1* and *Ccnb2*) regulation in the context of *Alkbh5* depletion, we assessed the turnover of cyclin B1 and B2 mRNA during reprogramming of WT and KO *Alkbh5* MEFs. Our data showed that the stability of both cyclin B1 and B2 mRNA were further reduced in *Alkbh5* KO MEFs compared to WT control during reprogramming (Fig. 4D). The reduction in cyclin B1 and B2 mRNA stability in *Alkbh5* KO MEFs during reprogramming was reflected in the reduction of their expression at both the RNA and protein levels (Fig. 3D,E). Furthermore, we performed m6A immunoprecipitation (IP) on day 3 of reprogramming, and our data revealed increased m6A enrichment on both cyclin B1 and B2 mRNA in *Alkbh5* KO MEFs compared to WT MEFs (Fig. 4E). Taken together, our data suggest that increased m6A levels at cyclin B1 and B2 mRNA results in decreased stability, hence reduced expression at both the RNA and protein level, leading to cell cycle arrest at G2/M phase and a reduction in reprogramming efficiency.

### ALKBH5 overexpression in the late phase enhances reprogramming efficiency by upregulating *Nanog*

**Fig. 5.**
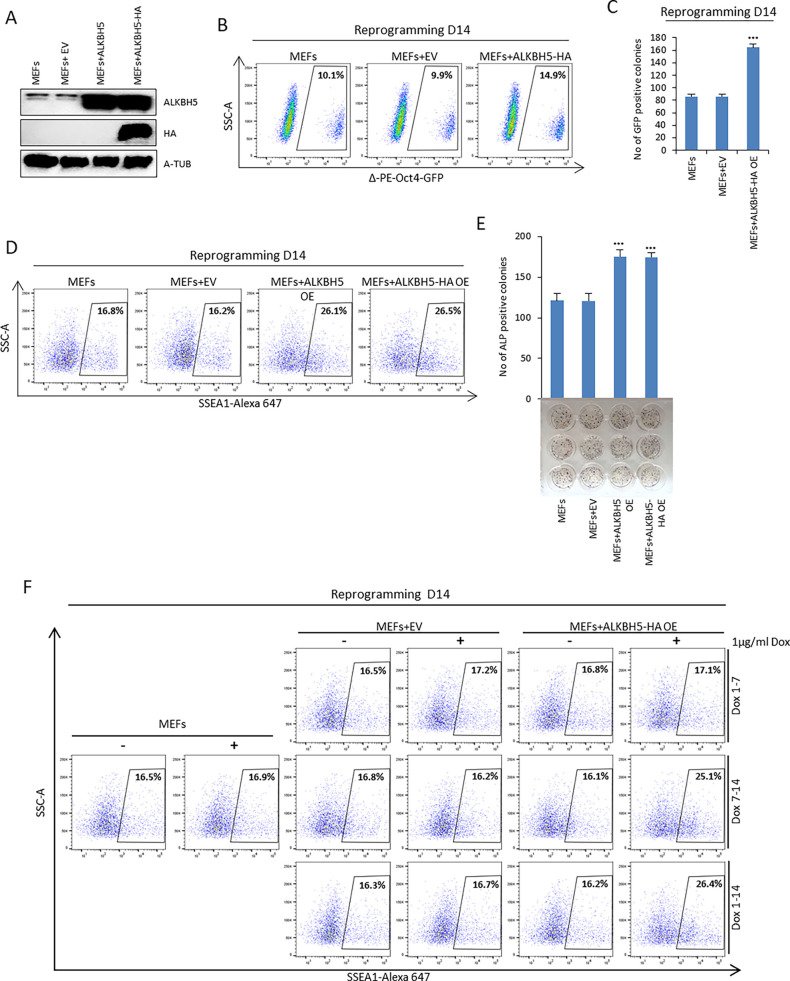
**ALKBH5 overexpression enhances reprogramming efficiency.** (A) Immunoblot analysis of ALKBH5 protein levels after lentiviral infection of MEFs with empty vector, ALKBH5 or ALKBH5 tagged with HA (ALKBH5–HA). α-tubulin (A-TUB) was used as a loading control. (B) Fraction of Δ-PE-Oct4-GFP-positive cells at day 14 of reprogramming of MEFs, MEFs with empty vector (EV) and MEFs overexpressing ALKBH5–HA determined by FACS analysis. (C) Number of Δ-PE-Oct4-GFP-positive colonies on day 14 of reprogramming. (D) Fraction of SSEA1-positive cells at day 14 of reprogramming of MEFs, MEFs with empty vector (EV) and MEFs overexpressing ALKBH5 or ALKBH5–HA as determined by FACS. (E) Reprogramming efficiency in MEFs overexpressing ALKBH5 or ALKBH5–HA was assessed by counting the number of ALP-positive colonies on day 14 of reprogramming. (F) Fraction of SSEA1-positive cells determined by FACS analysis on day 14 of reprogramming. Temporal overexpression of ALKBH5–HA by 1 µg/ml doxycycline (Dox) was carried out from day 1 to day 7, day 7 to day 14 or day 1 to day 14. MEFs were used as a negative control. Images in A, B, D and F are representative of three repeats. Quantitative data are shown as the mean±s.d.; *n*=3. ***P<0.001 (paired Student's *t*-test).

We assessed the impact of ALKBH5 overexpression on the reprogramming process. We used lentiviral expression to achieve high expression of both ALKBH5 and ALKBH5–HA during reprogramming (Fig. 5A). Our data revealed that overexpression of ALKBH5–HA enhanced the reprogramming efficiency by increasing the fraction of Δ-PE-Oct4-GFP-positive cells and the number of GFP-positive colonies as compared to the control (Fig. 5B,C). We further confirmed our data using flow cytometry to assess the fraction of SSEA1-positive cells and ALP-positive colonies. In agreement with our previous data (Fig. S3V), overexpression of either ALKBH5 or ALKBH5–HA enhanced the reprogramming process, as measured by an increase in the percentage of SSEA1-positive cells and an increase in the number of ALP-positive colonies at day 14 of reprogramming (Fig. 5D,E). Moreover, overexpression of ALKBH5 and ALKBH5–HA increased both the E-Cad single-positive population and the Δ-PE-Oct4-GFP and E-Cad double-positive population compared to the control (Fig. S5A). In addition, the percentage of the Δ-PE-Oct4-GFP-positive cells gated on the SSEA1-positive population increased upon overexpression of either ALKBH5 or ALKBH5-HA (Fig. S5B).

To investigate at what time ALKBH5 overexpression enhances reprogramming efficiency, we used a Dox inducible overexpression system. We did not find any significant effect of ALKBH5 overexpression on the reprogramming efficiency at the early phase from day 1 to day 7. However, the percentage of SSEA1-positive cells at day 14 was greatly increased after overexpression of ALKBH5–HA from day 1–14, as well as after overexpression from day 7–14 only (Fig. 5F). To determine the precise time point at which ALKBH5 overexpression has a positive impact on the reprogramming process, we used Dox-inducible overexpression of ALKBH5–HA in Δ-PE-Oct4-GFP MEFs to induce ALKBH5 expression at seven different time intervals (day 0–2, 2–4, 4–6, 6–8, 8–10, 10–12 and 12–14), and we estimated the reprogramming efficiency using Δ-PE-Oct4-GFP by flow cytometry on day 14. In agreement with our previous data (Fig. 5F and Fig. S2D), ALKBH5-HA overexpression did not have any impact on the early phase of reprogramming, while the positive impact was observed from day 8 onwards (Fig. S5C).

**Fig. 6.**
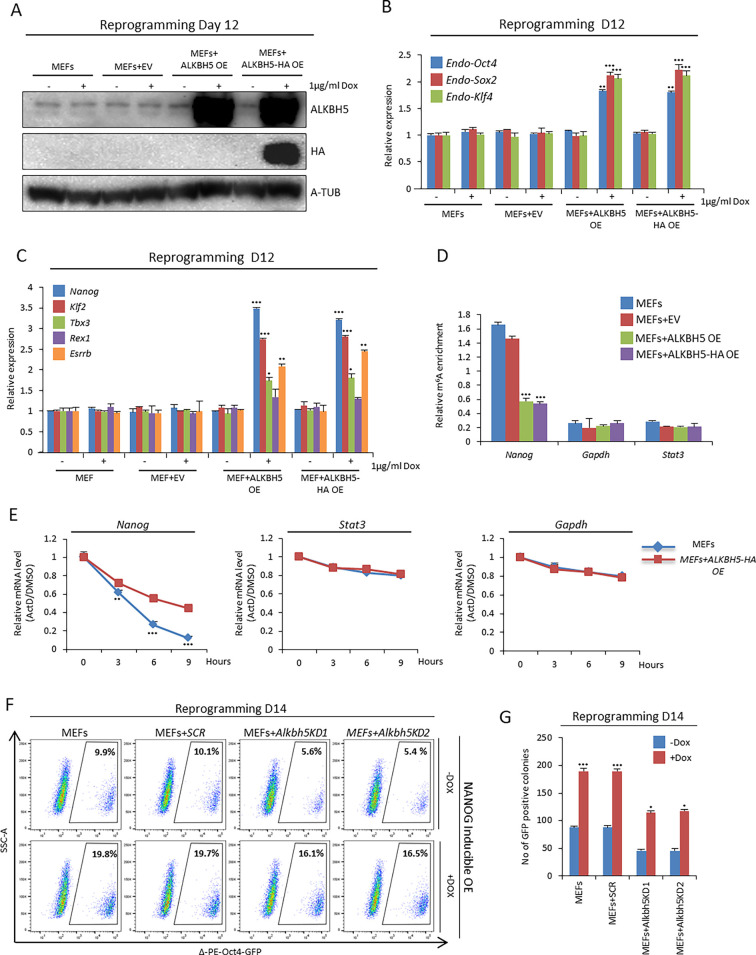
**ALKBH5 overexpression in the late phase of reprogramming stabilizes *Nanog* transcripts, resulting in increased *Nanog* expression.** (A) Immunoblot analysis of ALKBH5 protein levels on day 12 of reprogramming. MEFs and lentivirus-infected MEFs with empty vector (EV), ALKBH5 or ALKBH5–HA were treated with or without Dox (1 µg/ml) on day 10, and cells were harvested on day 12. α-tubulin (A-TUB) was used as a loading control. (B) Endogenous expression of pluripotency factors (*Oct4, Sox2* and *Klf4*) in reprogrammed MEFs on day 12. MEFs and lentivirus-infected MEFs with empty vector, ALKBH5 or ALKBH5–HA were treated with or without Dox (1 µg/ml) on day 10, and cells were harvested on day 12 and analyzed by qPCR. The data were normalized to the housekeeping gene *Gapdh*. (C) Expression of pluripotency markers in reprogrammed MEFs on day 12. MEFs and lentivirus-infected MEFs with empty vector, ALKBH5 or ALKBH5–HA were treated with or without Dox (1 µg/ml) on day 10, and cells were harvested on day 12 and analyzed by qPCR. The data were normalized to the housekeeping gene *Gapdh*. (D) m^6^A-IP qPCR data of *Nanog*, *Gapdh* and *Stat3* from reprogrammed MEFs on day 12. MEFs were infected with empty vector, or were MEFs overexpressing ALKBH5 or ALKBH5–HA, and were analyzed on day 12 of reprogramming. m^6^A qPCR data were normalized to the inputs. (E) Stability of *Nanog* transcripts in reprogrammed MEFs as a control, or MEFs with ALKBH5–HA overexpression, on day 12 of reprogramming. Actinomycin D (ActD) was added on day 12. Cells were treated with either DMSO or 5 µM ActD at different time points from 0 to 9 h. *Gapdh* and *Stat3* were used as negative controls, and the data of cells treated with 5 µM ActD were normalized to that from DMSO-treated cells. (F) Fraction of Δ-PE-Oct4-GFP-positive cells on day 14 of reprogramming determined by FACS analysis using Dox-inducible overexpression of NANOG. Cells use were WT MEFs or lentivirus-infected MEFs with scrambled shRNA (SCR), or two different shRNAs targeting *Alkbh5* (KD1 and KD2). Reprogrammed cells were treated with or without Dox (1 µg/ml) from day 8. (G) Number of Δ-PE-Oct4-GFP-positive colonies on day 14 of reprogramming. Images in A and F are representative of three repeats. Quantitative data are shown as the mean±s.d.; *n*=3. *P<0.05, **P<0.01, ***P<0.001 (paired Student's *t*-test).

To investigate the molecular mechanism responsible for enhancing reprogramming efficiency upon overexpression of ALKBH5 at the late phase, we used a Dox inducible system for temporal overexpression of ALKBH5 from day 10 to day 12 (Fig. 6A,B). We found that overexpression of ALKBH5 resulted in upregulation of the endogenous RNA level of reprogramming factors such as *Oct4*, *Sox2* and *Klf4* and other pluripotency factors, including *Klf2*, *Tbx3* and *Esrrb*, and in particular *Nanog* (Fig. 6B,C). We obtained similar results upon overexpression of ALKBH5 from day 8 to day 10 (Fig. S6A–C). Previous studies have reported that *Nanog* is regulated post-transcriptionally in both mouse and human ESCs by the m^6^A machinery (Batista et al., 2014; Geula et al., 2015). We hypothesized that *Nanog* transcripts are post-transcriptionally regulated through the m^6^A modification during reprogramming and that overexpression of the m^6^A demethylase ALKBH5 will reduce m^6^A levels, potentially affecting the stability of *Nanog* transcripts. To test this hypothesis in the reprogramming context, we performed m^6^A IP at day 12 of reprogramming and indeed found that overexpression of ALKBH5 decreased the m^6^A level on *Nanog* transcripts (Fig. 6D). Furthermore, we assessed the stability of *Nanog* transcripts after overexpression of ALKBH5. We found that overexpression of ALKBH5 resulted in increased stability of *Nanog* transcripts (Fig. 6E). Next, we assessed whether ALKBH5 overexpression could rescue the *Alkbh5* KO phenotype in reprogramming. Our data revealed that overexpressing either ALKBH5 or ALKBH5–HA in *Alkbh5* KO MEFs could restore the reprogramming efficiency (Fig. S6D-G). Finally, we tested whether NANOG overexpression could compensate for *Alkbh5* knockdown. We used a Dox-inducible overexpression system to control NANOG overexpression during reprogramming (Fig. S6H). Our data revealed that overexpression of NANOG enhanced reprogramming in both WT MEFs and *Alkbh5* knockdown MEFs as measured on day 14 as an increase in the fraction of Δ-PE-Oct4-GFP-positive cells and the number of Δ-PE-Oct4-GFP-positive colonies (Fig. 6F,G). Furthermore, overexpression of NANOG resulted in an increased fraction of SSEA1-positive cells in both WT and *Alkbh5* KD MEFs at day 14 of reprogramming (Fig. S6I).

Taken together, our findings suggest that ALKBH5 overexpression in the late phase of reprogramming enhances reprogramming efficiency by decreasing the m^6^A level at *Nanog* transcripts, thus stabilizing these transcripts and resulting in upregulation of *Nanog*. In addition, overexpression of NANOG can compensate for the negative effect *Alkbh5* depletion has on reprogramming efficiency.

## DISCUSSION

Ectopic expression of the four transcription factors OCT4, SOX2, KLF4 and c-MYC (OSKM) in somatic cells can establish the pluripotency regulatory circuitry, resulting in massive changes at both the epigenetic and transcriptional levels and the generation of iPSCs (Takahashi et al., 2007; Takahashi and Yamanaka, 2006). Successful therapeutic application of these iPSCs will likely require a comprehensive understanding of the molecular mechanism underlying somatic cell reprogramming. Here, we aimed to dissect the role of the m^6^A demethylase ALKBH5 in somatic cell reprogramming.

Our data revealed that the catalytic activity of ALKBH5 is required for the regulation of the reprogramming process. Both catalytically inactive ALKBH5 (H205A) and catalytically deleted ALKBH5 (CD) overexpression failed to restore the reduced reprogramming efficiency in *Alkbh5* KO MEFs, and their overexpression in WT MEFs impaired the reprogramming efficiency and cell proliferation.

Resetting the pluripotency cell cycle pattern is an essential step in achieving successful iPSC generation, suggesting that the cell division rate is a key parameter for somatic cell reprogramming (Hanna et al., 2009). In agreement with that, *Tp53* and *Ink4/Arf* (*Cdkn2a*) have been shown to act as barriers to the reprogramming process (Kawamura et al., 2009; Hong et al., 2009; Li et al., 2009). Additionally, G2/M cell cycle regulators have been reported to maintain pluripotency, and the Cdk1–cyclin B1 complex has been reported to enhance the reprogramming process (Gonzales et al., 2015; Wang et al., 2017). Moreover, the m^6^A machinery has been reported to be involved in regulating Cdk1 and cyclin B2, and knockout of *Fto* results in decreased expression of Cdk1 and cyclin B2, causing G2/M cell cycle arrest in spermatogonia (Huang et al., 2019). Here, we showed that *Alkbh5* depletion in MEFs or during the early phase of somatic cell reprogramming decreased the expression of cyclin B1 and B2, accompanied by cell cycle arrest at G2/M phase, which in turn resulted in reduced proliferation and MET transformation rate, ultimately leading to impaired reprogramming efficiency. Overexpression of either cyclin B1 or B2, or both, restored the phenotype of *Alkbh5* depletion and additionally enhanced the reprogramming process. However, we do not rule out that other mechanisms might also be at play; for example, we acknowledge that we cannot formally exclude the possibility that *Alkbh5* might have a direct effect on MET. This could be an interesting point for future studies. Furthermore, future work, including m6A-IP-seq, could provide new candidates downstream of ALKBH5 that might overlap with the functional role of cyclin B1 or B2 in regulating G2/M phase during reprogramming.

Moreover, in contrast with what was observed for the early phase of reprogramming, we found that depletion of *Alkbh5* in the late phase of reprogramming did not have a significant effect on reprogramming efficiency. This indicates that the negative effect of *Alkbh5* depletion on reprogramming efficiency occurs specifically during the early phase, where both resetting of the cell cycle pattern and morphological transformation to epithelial-like cells occur.

Recent studies have revealed that the m^6^A modification on mRNA is essential in regulating pluripotency, self-renewal of stem cells, somatic cell reprogramming and early embryonic development (Chen et al., 2015; Aguilo et al., 2015). Regulation of pluripotency by the m^6^A machinery has been reported in both mouse and human ESCs, where *Mettl3* and/or *Mettl14* depletion induces a hyper-pluripotent state, presumably through increasing the m^6^A level at several pluripotency related transcripts, such as *Nanog*, resulting in increased transcript stability that hinders cells from exiting the pluripotency state (Geula et al., 2015; Batista et al., 2014). NANOG is a key regulator of pluripotency and is required for acquiring pluripotency during the late phase of reprogramming (Pan and Thomson, 2007; Silva et al., 2009). A synergistic role of NANOG in overexpression together with DNA demethylation agents in the late phase of reprogramming has been reported to enhance acquisition of the pluripotency state (Theunissen et al., 2011; Silva et al., 2009). Moreover, NANOG co-binds with OCT4, SOX2 and KLF4 to many regulatory regions to facilitate the binding of the coactivator P300 (also known as EP300) (Chen et al., 2008). Here, we showed that ALKBH5 overexpression in the late phase of reprogramming decreases the m^6^A level at *Nanog* transcripts, resulting in increased *Nanog* stability and enhanced reprogramming efficiency. Overexpression of NANOG enhances the reprogramming efficiency in both WT and *Alkbh5* knockdown MEFs. Consistent with our findings, ALKBH5 has been reported to positively regulate *Nanog* stability and expression in response to hypoxia-inducible factor (HIF)-1α and HIF-2α in breast cancer stem cells (BCSCs) (Zhang et al., 2016).

A recent study reported that YTHDF2 and YTHDF3, but not YTHDF1, regulates MET events in somatic cell reprogramming in an m^6^A-dependent manner through the Hippo signaling pathway effector *Tead2* (Liu et al., 2020). Other studies have shown redundancy among the three paralogs *Ythdf1*, *Ythdf2* and *Ythdf3*, suggesting that they can have adequate functional compensation, at least in some biological contexts (Zaccara and Jaffrey, 2020; Lasman et al., 2020). It would be interesting to assess the role of *Ythdf1*, *Ythdf2* and *Ythdf3*, as well as any redundancy, in the context of *Alkbh5* depletion in future studies.

**Fig. 7.**
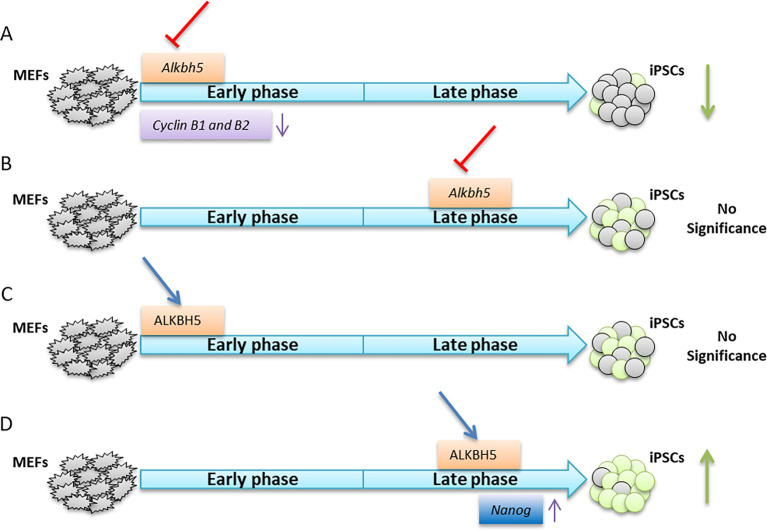
**Model showing the biphasic role of ALKBH5 in somatic cell reprogramming.** (A) Depletion of *Alkbh5* specifically in the early phase of reprogramming decreases the reprogramming efficiency by reducing the expression of cyclin B1 and B2. (B) Depletion of *Alkbh5* in the late phase of reprogramming has no impact on reprogramming efficiency. (C) Overexpression of ALKBH5 in the early phase of reprogramming does not affect the reprogramming efficiency. (D) Overexpression of ALKBH5 in the late phase enhances the reprogramming efficiency by increasing *Nanog* expression.

In conclusion, we provide mechanistic insight into the epitranscriptional regulation of somatic cell reprogramming by elucidating the biphasic regulatory role of ALKBH5 in modulating reprogramming efficiency at the post-transcriptional level in a stage specific manner (Fig. 7).

## MATERIALS AND METHODS

### MEFs derivation

The wild-type (WT), knockout (KO) *Alkbh5* and homozygous floxed *Alkbh5* MEFs (*Alkbh5^f^*^/*f*^) were all derived from embryos at 13.5 days post coitum (dpc.). Mice were housed and mated in Norwegian Transgenic Center (NTS). Briefly, pregnant C57BL/6 female mice were euthanized on 13.5 or and embryos were dissected. All animal experiments were performed according to approved guidelines. The internal organs, head, and limbs were removed and used for genotyping. Then the remaining tissues were trypsinized using 0.25% trypsin for 30 min at 37°C with shaking to make single-cell suspensions, then cells were pooled and plated in MEF medium (DMEM plus 10% FBS) until 80% confluence then trypsinized and stored in freezing solution (FBS+10% DMSO) in liquid nitrogen for future use. MEFs were cultured and maintained in Dulbecco's modified Eagle's medium (DMEM) plus 10% FBS (tetracycline free FBS PAN-Biotech Catalog # P30-2602TC) until reaching 70–80% confluence, then passaged at 10^5^ cells per well of six-well plate.

*Alkbh5^f^*^/f^ MEFs were plated at 10^5^ cells per well of six-well plate overnight. Next day, the cells were transfected with KA1153 pPB-CAG-MerCreMer-IN (Addgene plasmid #124183) together with PBase (Guo et al., 2009), and PB-CAG-HA-IRES-Puro (a kind gift from Prof. Hitoshi Niwa, Kumamoto University IMEG, Japan) using Lipofectamin 2000 (Invitrogen #11668019) or Fugene 6 (Promega #E2691). The medium was changed after 5 h. Next day, the cells were cultured with medium containing 2 µg/ml puromycin (Thermo Fisher Scientific #A1113803) for 2 days. Then cells were treated with 1 µM of 4 hydroxytamoxifen (4-OH Tam) (Merck #H7904-5MG) for depletion of *Alkbh5* at indicated time points. Cells are routinely tested and are mycoplasma free.

### Reprogramming

For reprogramming, dissociated MEF cells at early passages were plated at 10^5^ cells per well of a six-well plate or 5×10^5^–6×10^5^ cells per 10 cm dish depending on the purpose of experiment. The cells were infected with equal ratio of the retroviruses expressing the four reprogramming factors (OCT4, SOX2, KLF4 and c-MYC) and incubated at 37°C for 8–12 h with 8 µg/ml of polybrene. The medium was changed next day. For either knockdown or overexpression experiments during reprogramming, the MEFs were plated at 10^5^ cells per well of a six-well plate, and infected with lentivirus for 8 h, them medium was changed, and next day the selectable markers were added for 2 days. If the cells were trypsinized at day 7 of reprogramming, the reprogrammed cells were cultured with feeder layer CF-1 MEFs Irradiated, P3 2M (AMS biotechnology #GSC-6201G 2M or #GSC-6101G 7M) and LIF ESGRO^®^ recombinant mouse LIF protein (1000 units/ml) (Millipore #ESG1107). For induction of the transgene, Stemolecule doxycycline hyclate 10 mg (Stemgent#04-0016) was added at 1 µg/ml every 2 days. OG2 MEFs were a kind gift from Professor Hans R. Schöler (Department of Cell and Developmental Biology, Max Planck Institute for Molecular Cell Biology and Genetics; Velychko et al., 2019). The reprogramming efficiency was checked on day 14 by flow cytometry.

The Piggybac reprogramming protocol was used as previously described (Kim et al., 2015). Briefly MEFs were seeded in 10^5^ cells per 10 cm dish (multiple dishes were used in parallel) overnight. The next day, a mixture of 500 ng of PB-TAC-OSKM vector obtained from Addgene (plasmid #80481), 500 ng of pPB-CAG-rtTA-IN (Addgene plasmid #60612), and 1000 ng of piggybase plasmid at a Fugene/DNA ratio of 4 μl:1 μg DNA. Next day, the medium was changed for medium with dox at a concentration of 1 µg/ml for 1 day. The next day, cells were checked for mCherry positivity and 10^4^ cells were seeded in multiple wells of a six-well plate. The reprogrammed cells were checked by flow cytometry at day 7 and day 14 for both mCherry and SSEA1–Alexa-Fluor-488.

Both WT and KO *Alkbh5* MEFs were seeded at 10^5^ cells per well of a six-well plate and infected with equimolar ratio of OSKM retrovirus and lentivirus for expression of both FUW-M2rtTA (Addgene plasmid #20342) and FUW-TetO-Nanog (Addgene plasmid #40800). On day 7 of reprograming, dox was added at concentration of 1 µg/ml, and cells were checked for reprogramming efficiency at day 14 by flow cytometry.

### Retrovirus preparation

Plate E cells were used for preparation of retrovirus (Cell bio labs #RV-101). Plate E cells were plated at 10^6^ cells per 10 cm dish in DMEM plus 10% FBS (tetracycline free FBS PAN-Biotech Catalog #P30-2602TC) until reaching 70–80% confluence. Then cells were transfected with 9 µg of each of pMXs-Oct4 (Addgene plasmid #13366), pMXs-Sox2 (Addgene plasmid #13367), pMXs-Klf4 (Addgene plasmid #13370), pMXs-c-Myc (Addgene plasmid #13375) per 10 cm dish using Fugene 6 (Promega cat. no. #E2691), and the medium was changed after 8 h using Iscove's modified Dulbecco's medium (IMDM) plus 10% FBS. Retroviral supernatant were harvested after 48 and 72 h and centrifuged at 177 ***g*** for 5 min at 4°C. The retroviral supernatant was used freshly or frozen in aliquots at −80°C. The viral titer was estimated to produce up to 7–8% SSEA1 on day 7 of reprogramming; use of the GFP control showed that there was >85% infection efficiency by FACS.

### Lentivirus preparation

Lenti-X 293T cells were used for preparation of lentivirus (Takahara Clontech #632180). Lenti-X 293T cells were plated at 10^6^ cells per 10 cm dish in DMEM plus 10% FBS (tetracycline free FBS) until the cells reached 70–80% confluence. The cells were transfected with PsPAx2 (Addgene plasmid #12260), pMD2.G (Addgene plasmid #12259), and the vector encoding either shRNA for knockdown *Alkbh5* or overexpression of ALKBH5, ALKBH5–HA, ALKBH5–HA (H205A), ALKBH5–HA (CD), NANOG, or cyclin B1 or B2 using Fugene 6. The medium was changed after 8 h using IMEDM plus 10% FBS. Lentiviral supernatants were harvested after 48 and 72 h and centrifuged at 177 ***g*** for 5 min at 4°C.The lentiviral supernatant was used freshly or frozen in concentrated aliquots using Lenti-X™ concentrator (Takahara, cat. number #631232) and stored at −80°C.

### Cell proliferation assay

MEFs were plated at 10^4^ per well of 24-well plates in quadruplicate. Then, at each indicated time point, four wells were trypsinized and counted independently using Life Technologies #C10228 Countess™ Cell Counting Chamber Slides. Medium was replaced every 2 days and the data are presented as mean±s.d. for quadruplicate samples.

For the reprogramming experiment, MEFs were plated at 1×10^5^ cells per well of 6-well plate in triplicate, and infected with equal molar ratio of retroviral titer encoding Oct4, Sox2, Klf4, and c-Myc with or without pMXs-DsRed was obtained from Addgene (plasmid #22724) as a control for 6 h then medium was changed. At 8 h after infection, cells were treated with either ethanol or 1 µM 4-OH-Tam for depletion of *Alkbh5*. Cells were trypsinized at indicated time points and counted. Medium was replaced every 2 days, and the data are presented as mean±s.d. for triplicate samples.

### Genotyping

Cells from tissue biopsies were suspended in lysis buffer (1 M Tris-HCl pH 8, 5 M NaCl, 0.5 M EDTA and 10% SDS) and Proteinase K (20 mg/ml; Thermo Fisher Scientific #25530031) was freshly added and incubated at 37°C for 4 h to overnight. Then 300 µl of 5 M NaCl was added followed by vortexing and incubation on ice for 10 min then spinning at 112 ***g*** for 4 h. Then the supernatant was removed and transferred to new tube followed by 650 µl Iso-propanol and vortexing, and incubation at RT for 15 min, then centrifugation at 21,130 ***g***. Then the supernatant was discarded and the pellet was dissolved in 200 µl TE buffer, followed by incubation at 55°C for 10 min, then the DNA concentration is measured and 10–50 ng was used per reaction.

### Cloning

Both mALKBH5 and mALKBH5–HA were amplified from the cDNA using gateway forward and reverse primer using PrimeSTAR GXL DNA polymerase (Takahara Clontech #R050A-TAK). The PCR product was purified using QIAquick PCR Purification Kit (Qiagen #28106), then shuttled to Gateway™ pDONR™221 Vector (Invitrogen #12536017) using Gateway™ BP Clonase™ II Enzyme mix (Invitrogen #11789020). Then the construct was transformed to One Shot™ Stbl3™ Chemically Competent *E. coli* (Thermo Fisher Scientific #C737303). Positive clones were screen by colony PCR and restriction digestion then positive colonies were sent for sequencing. The correct clone was used as entry clone and then the construct was shuttled to destination vector pLX301 (Addgene plasmid #25895) For constitutive overexpression of either ALKBH5 or ALKBH5-HA, and pCW57.1 (Addgene plasmid #41393) for dox-inducible overexpression using LR clonase (Thermo Fisher Scientific #11791020) based on the manufacturer's protocol. 2 μl of Gateway reaction was used for transformation of Stbl3 competent cells. Then colonies were screened by colony PCR and restriction digestion. The positive colonies were sent for sequencing and the correct colony was propagated and the plasmids were purified using Qiagen (Endotoxin free kit #12362), and used for making the virus.

### shRNA cloning

Two shRNAs for targeting mouse *Alkbh5* (Table S1) were annealed in annealing buffer by heating for 10 min at 95°C in thermocycler then cooling by gradual decreasing the temperature to 4°C for 30 min. Then the annealed oligonucleotides were ligated using T4 DNA Ligase (5 U/µl) (Thermo Fisher Scientific #EL0011) to either pLKO.1 puro (Addgene plasmid #8453) for constitutive knockdown or Tet-pLKO-puro (Addgene plasmid #21915) for dox inducible knockdown which was linearized with AgeI-HF (NEB #R3552L) and EcoRI-HF (NEB #R3101S) restriction enzymes. Then the ligated product was transformed to One Shot™ Stbl3™ Chemically Competent *E. coli* (Thermo Fisher Scientific #C737303). Several colonies were picked up and sent for sequencing. The positive clones were propagated and the plasmid was purified using Qiagen (Endotoxin free kit #12362) and used for making the virus.

Both pENTR vector encoding both cyclin B1 (Addgene plasmid #136340) and B2 (Addgene plasmid #136341) were obtained from Addgene. Then the construct was shuttled through gateway cloning system using LR clonase into pMXs-GW (Addgene plasmid #18656) retroviral vector. Then several colonies were picked up and correct colonies were confirmed by colony PCR and sequencing. Then positive colony was propagated and the plasmids were purified using Qiagen (Endotoxin free kit #12362), and used for making the retrovirus.

The pDONR™221 vector encoding *mAlkbh5-HA* tag used as template for making both point mutation ALKBH5–HA(H205A) and catalytic deletion ALKBH5–HA (CD), using a combination of overlap extension PCR and the Q5^®^ Site-Directed Mutagenesis Kit (NEB# E0554S) with the primers listed in Table S1, based on the manufacturer's protocol with some modifications. Positive colonies were confirmed by colony PCR and sequencing. Then positive colony was used to shuttle the construct to either to destination vector pLX301 or pCW57.1 using LR clonase based on the manufacturer's protocol. Then positive colony was propagated and the plasmids were purified using Qiagen (Endotoxin free kit #12362), and used for making the lentivirus.

### qPCR

TRIzol™ LS Reagent (Thermo Fisher Scientific #10296010) was used for RNA extraction according to the manufacturer’s protocol, then the RNA was dissolved in UltraPure™ DNase/RNase-free distilled water (Thermo Fisher Scientific #10977049), then 1 µg was used to make the cDNA using SuperScript™ IV VILO™ Master Mix with ezDNase™ enzyme (Thermo Fisher Scientific #11766050) based on the manufacturer's protocol. For real-time qPCR, 2 µl of cDNA was used per reaction using PowerUp™ SYBR™ Green Master Mix (Thermo Fisher Scientific #A25777). The transcript level was normalized to the internal control**.** Primers used are listed in Table S1.

### RNA stability

Cells were treated with 5 µg/ml of actinomycin D (Tocris #1229) for 3, 6 or 9 h. Total RNA was extracted at each time point. DMSO-treated cells was used as a control, and relative RNA expression was detected by qPCR.

### m6A dot blot

Total RNA was extracted from cells using TRIzol™ LS Reagent (Thermo Fisher Scientific #10296010) or RNeasy Plus Mini Kit (Qiagen #74134). mRNA was isolated and purified using the Dynabeads™ mRNA purification kit (for mRNA purification from total RNA preperations; Invitrogen #61006) following the manufacturer's instructions. For the m6A dot blot, mRNA was hybridized onto the Hybond-N+ membrane (GE Healthcare). After crosslinking spotted mRNA to the membrane using a Stratalinker 2400 UV Crosslinker, the membrane was blocked with 5% skimmed milk for 1 h, and incubated with mouse anti-m6A antibody (1:1000, Millipore #MABE1006) at 4°C overnight. Then the membrane was incubated with horseradish peroxidase (HRP)-conjugated donkey anti-mouse-IgG at room temperature for 1 h. The membrane was photographed using the ECL imaging system (Bio-Rad). Finally, the membrane was stained with 0.02% Methylene Blue. The relative m6A level was quantified using ImageJ.

### m6A IP-qPCR

Control and Alkbh5–HA-overexpressing reprogrammed cells at day 12 of reprogramming were harvested, and mRNA was extracted as described above. 1 to 2 µg of mRNA was fragmented at 70°C for 4 min. The mRNA was precipitated and the pellet was dissolved in Ultrapure DNase/RNase-free water, then incubated with pre-conjugated m6A/protein G beads (Dynabeads™ Protein G for Immunoprecipitation, #10003) in IP buffer, and incubated at 4°C for overnight. The mRNA was isolated from the beads using Trizol LS, and the RNA was used to make cDNA using SuperScript™ IV VILO™ Master Mix with ezDNase™ Enzyme (Thermo Fisher Scientific #11766050) based on the manufacturer's protocol. The m6A mRNA level was finally determined by real-time qPCR relative to the input.

### Western blotting

Cells were washed twice with ice-cold 1× PBS, and then scrapped and transferred to 1.5 ml Eppendorf tubes, then centrifuged (177 ***g*** for 5 min) and supernatant was discarded. The cells were lysed on RIPA lysis buffer (20 mM Tris-HCl PH7.5, 1 mM MgCl_2_, 500 mM NaCl, 20% glycerol, 0.5% NP-40, 1 mM EDTA, 1 mM EGTA) and freshly added 1× Halt protease inhibitor cocktail (100×) (Thermo Fisher Scientific #87786) and incubated on ice for 30 min. The lysed cells centrifuged at maximum speed (177 ***g***) for 30 min and the supernatant was transferred to new Eppendorf tubes. Then protein content was measured using Bradford protein assay (BSA) method, and then equal amounts of protein were lysed with 1x Bolt™ LDS Sample Buffer (Thermo Fisher Scientific B0008) and 1× Bolt™ Sample Reducing Agent (Thermo Fisher Scientific B0009). The sample was loaded on Bolt ready-made gels (4–12%) and transferred to PVDF or nitrocellulose Bio-Rad pads using the Trans-Blot Turbo Transfer System. Then the membrane was blocked using 5% skimmed milk in 1× Tris-buffered saline with 0.1% Tween 20 (TBST) buffer and then incubated with the primary antibody overnight. Next day, the membrane was washed three times using 1× TBST buffer and incubated with the secondary antibody for 1 h at room temperature. The membrane was washed three times using 1× TBST buffer, and then the protein detected with Pierce™ ECL Western Blotting Substrate (Thermo Fisher Scientific #32209) or SuperSignal™ West Femto Maximum Sensitivity Substrate (Thermo Fisher Scientific #34094), using Bio-Rad ChemiDoc XRS, and Precision Plus Protein™ Dual Color Standards (Bio-Rad #161-0374) as protein standards. Antibodies used are shown in Table S2. The raw data of western blots are included in Fig. S7.

### ALP staining

ALP staining was undertaken by using the Leukocyte Alkaline Phosphatase Kit (Sigma 85L3R) based on the manufacturer's protocol as previously described (Khodeer and Era, 2017).

### Cell cycle analysis

The cells were trypsinized and washed twice with 1× PBS. Then the cells was suspended in 300 µl ice-cold 1× PBS and 700 µl of ice-cold 100% ethanol was added drop by drop with vortexing. The cells were incubated at 4°C for at least 30 min. Then the cells were centrifuged and the pellet was suspended in 200 µl propidium iodide (PI)/RNase Staining Solution (Cell signaling 4087S) and incubated at room temperature for 30 min before analysis by FACS.

### Apoptosis

Detection of apoptotic cells was performed by using an FITC-Annexin V Apoptosis Detection Kit with 7-AAD (Biolegend #640922). Briefly, the cells were collected and washed twice with 1× PBS. The cells were suspended in 200 µl 1× binding buffer, 1 µl Annexin V–FITC and 7AAD (1:200). The cells were incubated at room temperature for 30 min in the dark. The cells were centrifuged (177 ***g*** for 5 min) and suspended in 300 µl 1× binding buffer and then analyzed by FACS.

### SSEA1 staining

Cells at indicated time points were washed two times with 1× PBS then trypsinized. The cells were counted and 1×10^6^ cells were washed again with 1× Hanks buffer and stained with 5 µl of Alexa Fluor^®^ 647 anti-mouse/human CD15 (SSEA-1) antibody (Biolegend #125608) or SSEA1–Alexa-Fluor-488 (Biolegend # 125610) in100 µl BD Pharmingen™ Stain Buffer (FBS) (BD Biosciences #554656) for 30 min on ice. The cells were washed once with 1× Hanks buffer and stained with 7AAD (1:200). The SSEA1-positive fraction was analyzed using FACS BD Fortessa. For the MET checking, the cells at indicated time points were washed two times with 1× PBS then trypsinized. The cells were counted, and 10^6^ cells were washed again with 1× Hanks buffer and double stained with either Thy1–Pacific Blue (Biolegend #140306) and E-Cad–Alexa-Fluor-647 (Biolegend #147308) or PDGFRβ–APC (Biolegend #136008) and E-Cad–Brilliant Violet 421 (Biolegend #147319).

### BrdU incorporation assay

The APC BrdU Flow Kit (BD Biosciences #552598) was used according to the manufacturer's protocol. Briefly, cells were labeled by adding 10 μM of BrdU to the culture medium. Treatment was performed for 1 h and then the cells were fixed and permeabilized. Then cells were treated with DNase for 1 h at 37°C. Then stained with anti-BrdU APC for 20 min at RT then resuspended in 7AAD and analyzed by FACS.

### Statistical analysis

All data were collected from at least three independent experiments. Data were analyzed using a paired Student's *t*-test or one-way ANOVA with Dunnett post-hoc test using Graphpad software. Significance is presented as *P<0.05, **P<0.01 and ***P<0.001. Error bars represented mean±s.d.

## Supplementary Material

Supplementary information

Reviewer comments
