# Supplementary material for: ALKBH5 regulates somatic cell reprogramming in a phase-specific manner
Source: J Cell Sci. 2022 Jun 10;135(11):jcs259824. doi: 10.1242/jcs.259824 (PMC9234673; doi:10.1242/jcs.259824)
Supplement: Supplementary information [file joces-135-259824-s1.pdf]

Supplementary Fig. 1

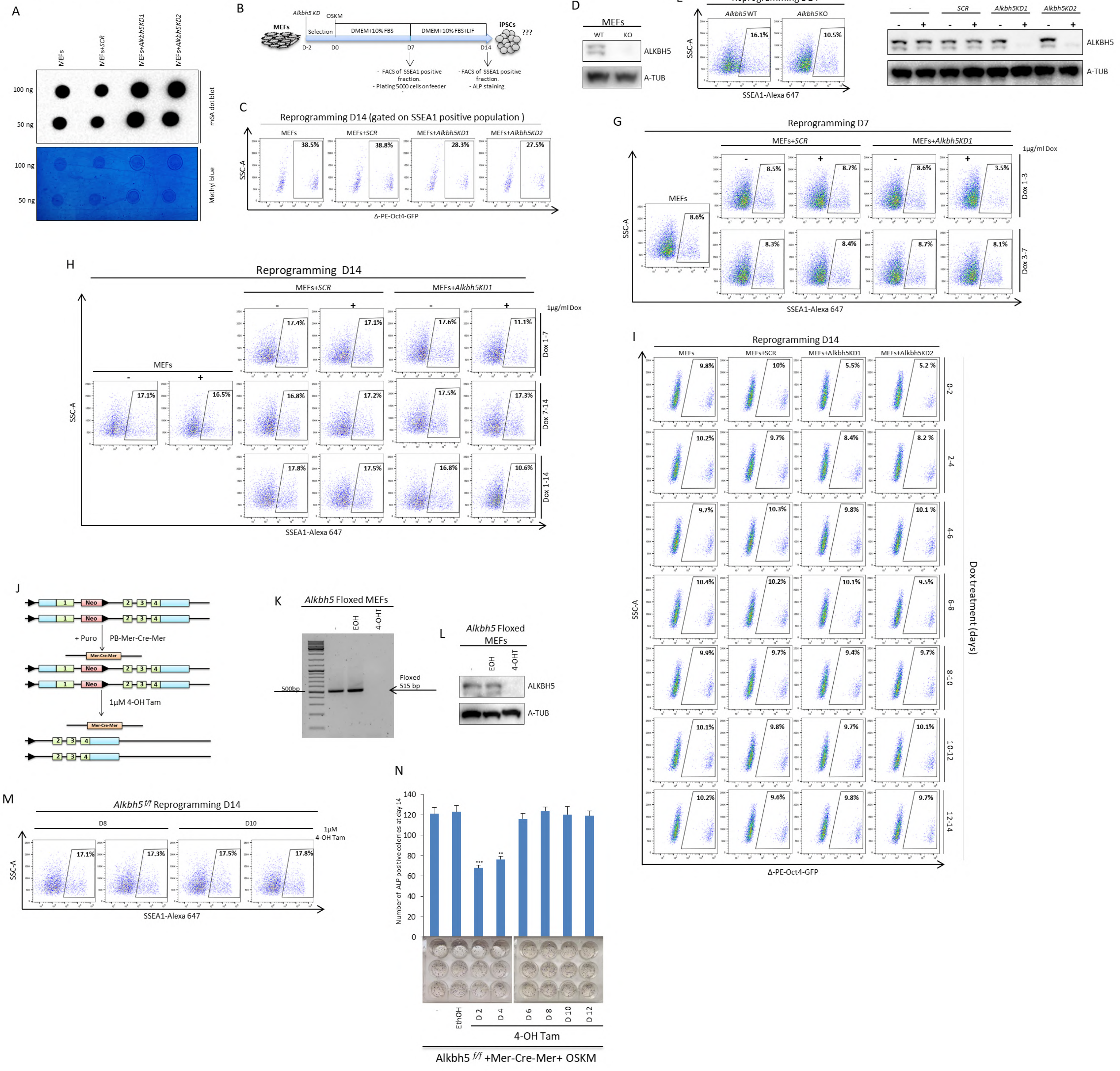

Journal of Cell Science • Supplementary information

Fig.S1. Depletion of Alkbh5 in the early stage impairs reprogramming efficiency.

(A) m^6A dot blot analysis of uninfected MEFs or MEFs infected either with lentiviral encoding scrambled shRNA (SCR) or two different shRNAs targeting Alkbh5 (upper panel). Methyl blue staining was used as a control to eliminate the difference in loaded mRNA amount (lower panel).

(B) Experimental design showing the timing of Alkbh5 knockdown, onset of reprogramming, and SSEA1 and ALP detection.

(C) Fraction of Δ-PE-Oct4-GFP-positive cells gated on the SSEA1-positive fraction determined by FACS analysis on day 14 of reprogramming.

(D) Immunoblot analysis of ALKBH5 protein levels in WT and Alkbh5 KO MEFs. A-TUB was used as loading control.

(E) Fraction of SSEA1-positive cells determined by FACS in WT and Alkbh5 KO reprogrammed MEFs on day 14 of reprogramming.

(F) Immunoblot analysis of ALKBH5 protein levels in MEFs infected with lentivirus encoding scrambled shRNA and two different shRNAs targeting Alkbh5. After selection with puromycin for 2 days, cells were treated with 1 μg/ml Dox to induce the expression of shRNA. A-TUB was used as a loading control.

(G) Fraction of SSEA1-positive cells determined by FACS in reprogrammed MEFs infected either with scrambled shRNA or shRNA targeting Alkbh5 with or without 1 μg/ml Dox treatment on day 7 of reprogramming. MEFs were used as a negative control.

(H) Fraction of SSEA1-positive cells determined by FACS in reprogrammed MEFs infected either with scrambled shRNA or shRNA targeting Alkbh5 with or without 1 μg/ml Dox treatment on day 14 of reprogramming. MEFs were used as a negative control.

(I) Fraction of the Δ-PE-Oct4-GFP-positive population was determined by FACS on day 14 of reprogramming throughout the whole reprogramming process. MEFs were infected either with scrambled shRNA or shRNA targeting Alkbh5 and treated with 1 μg/ml Dox every two days.

(J) Experimental design for Alkbh5 depletion. Homozygous $Alkbh5^{f/f}$ MEFs were derived from mice at 13.5 days post-coitum (d.p.c) before transfection with PB-GAG-Mer-Cre-Mer, selection with puromycin for 2 days, and treatment with 1 μM 4-OH Tam for induction of Cre to remove Alkbh5.

(K) Genotyping of homozygous $Alkbh5^{f/f}$ MEFs untreated or treated with either ethanol (negative control) or 1 μM 4-OH Tam for Alkbh5 removal. The band corresponds to the neomycin (Neo) PCR amplicon of 515 base pairs (bps).

(L) ALKBH5 immunoblot analysis of homozygous $Alkbh5^{f/f}$ MEFs untreated or treated with either ethanol (negative control) or 1 μM 4-OH Tam for Alkbh5 removal.

(M) Fraction of SSEA1-positive cells determined by FACS in reprogrammed MEFs on day 14 of reprogramming. Reprogrammed homozygous $Alkbh5^{f/f}$ MEFs treated with 1 μM 4-OH Tam for Alkbh5 depletion at day 8 or day 10 of reprogramming.

(N) Reprogramming efficiency as assessed by counting the number of ALP-positive colonies on day 14 of reprogramming. Reprogrammed homozygous $Alkbh5^{f/f}$ MEFs treated with 1 μM 4-OH Tam for Alkbh5 at days 2, 4, 6, 8, 10 and 12 of reprogramming, and ethanol treatment was used as a negative control. Data are shown as the mean ± SD; $n = 3$, $^*P < 0.05$, $^{**}P < 0.01$, and $^{***}P < 0.001$.

Journal of Cell Science • Supplementary information

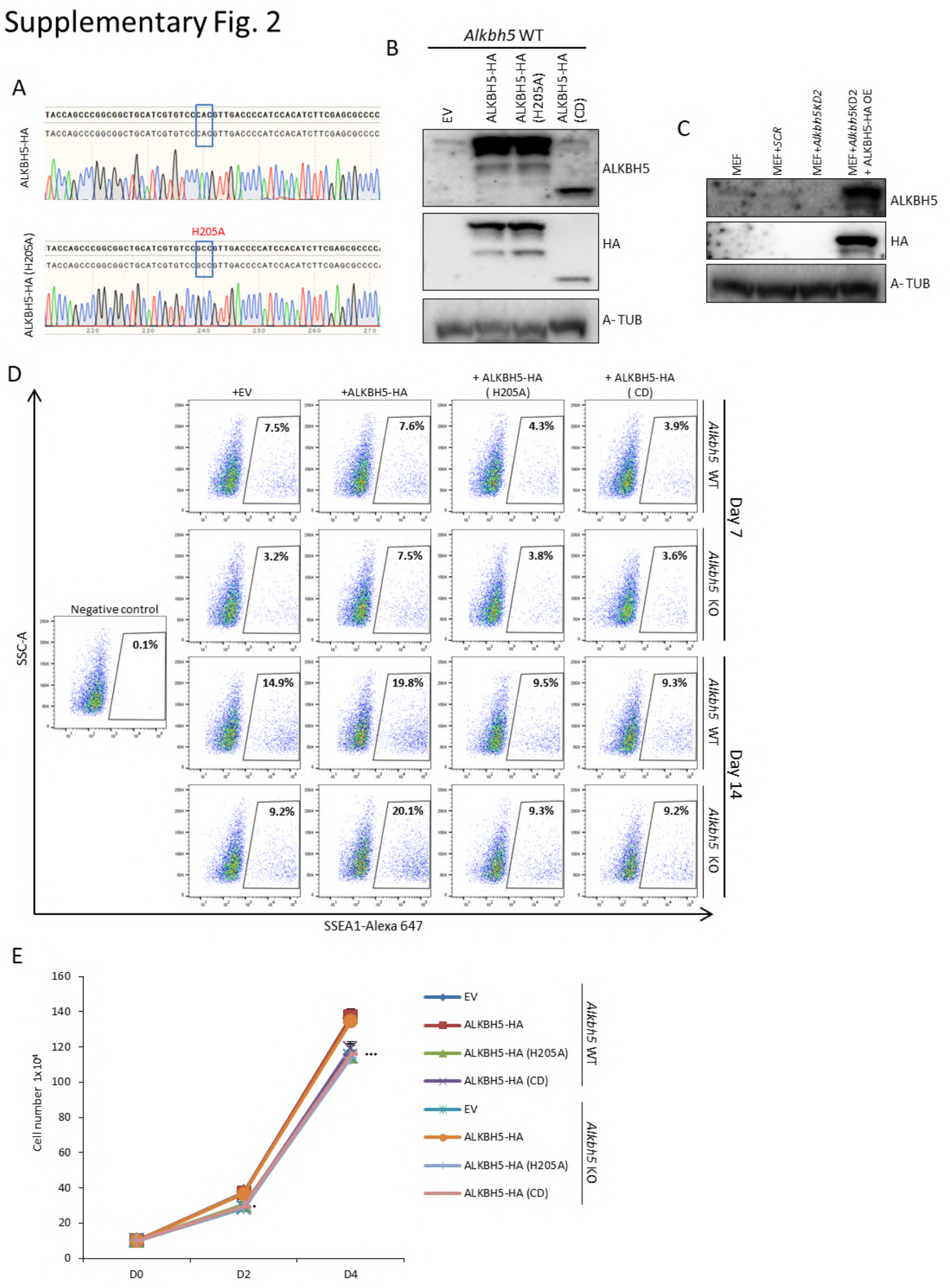

Fig. S2. ALKBH5 regulates somatic reprogramming through catalytic activity.

(A) Sequencing chromatogram of WT ALKBH5-HA and point mutated ALKBH5 (H205A); the blue box indicates the modified histidine to alanine amino acid.

(B) Immunoblot analysis of ALKBH5 in MEFs infected with empty vector (EV), ALKBH5-HA, ALKBH5-HA (H205A), or ALKBH5-HA (CD). A-TUB was used as a loading control.

(C) Immunoblot analysis of ALKBH5 in MEFs infected with either *SCR* shRNA, shRNA targeting the 3ÙTR of *Alkbh5* or shRNA targeting the 3ÙTR of *Alkbh5* and ALKBH5-HA. A-TUB was used as a loading control.

(D) Fraction of SSEA1-positive cells determined by FACS in reprogrammed MEFs on days 7 and 14 of reprogramming. Both WT and KO *Alkbh5* MEFs were infected with empty vector (EV), ALKBH5-HA, ALKBH5-HA (H205A), or ALKBH5-HA (CD). Unstained MEFs used as a negative control.

(E) Cell proliferation assay on day 4 of reprogramming. Both WT and KO *Alkbh5* MEFs were infected with empty vector (EV), ALKBH5-HA, ALKBH5-HA (H205A), or ALKBH5-HA (CD). Data are shown as the mean ± SD; n = 3, *P < 0.05, **P < 0.01, and ***P < 0.001.

Journal of Cell Science • Supplementary information

Supplementary Fig. 3

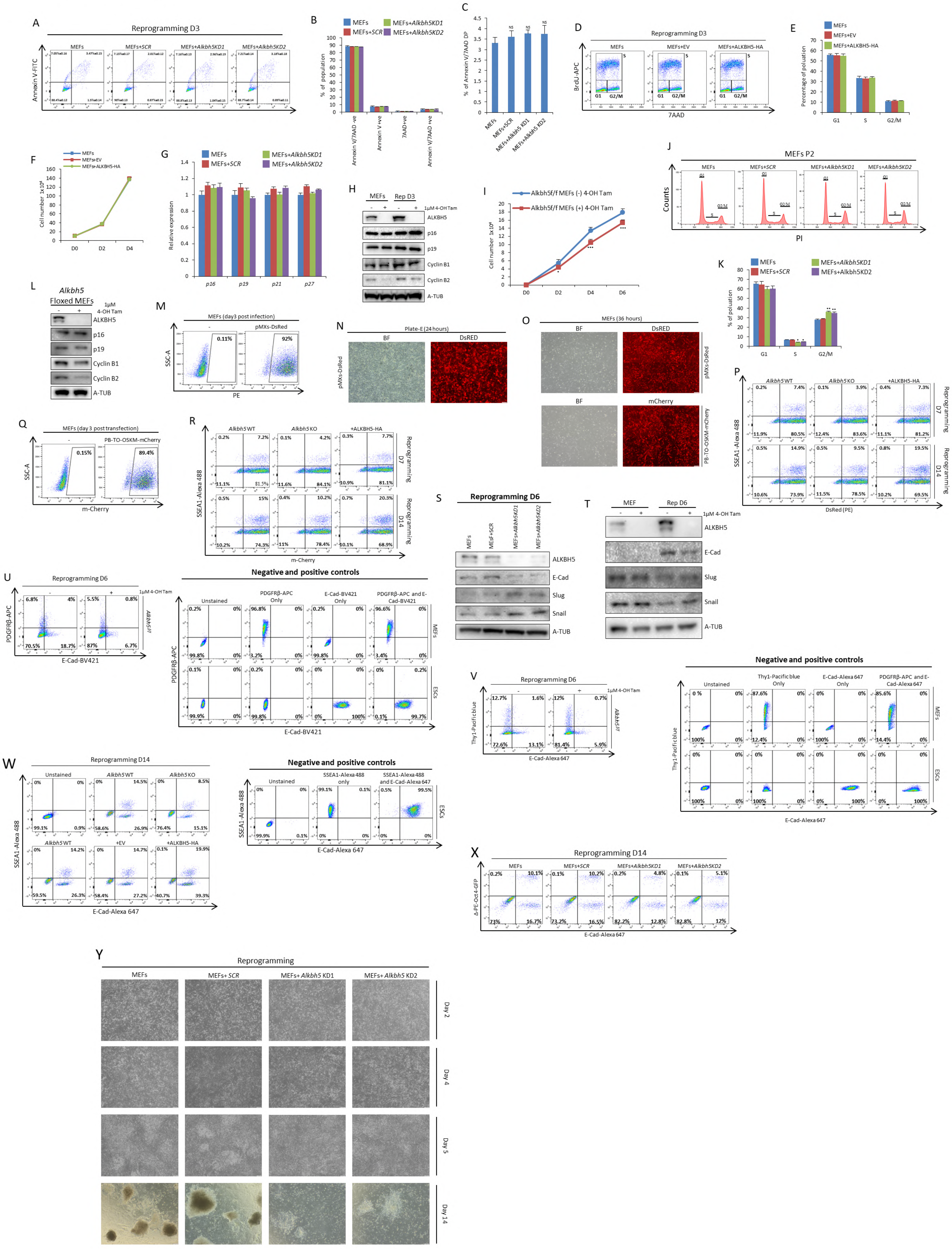

Journal of Cell Science • Supplementary information

Fig. S3. *Alkbh5* removal impairs cell reprogrammed proliferation in either MEFs or MEFs without increasing apoptosis.

(A) Fraction of apoptotic cells determined by FACS in reprogrammed MEFs uninfected or infected either by scrambled shRNA or two shRNAs targeting *Alkbh5* was assessed at day 3 of reprogramming using double staining with Annexin V and 7AAD staining.

(B) Analysis of cell apoptosis data determined by FACS in (Supplementary Fig. 3 A), each of 7AAD or Annexin V single positive (+ve) or negative (-ve), Annexin V/7AAD +ve or Annexin V/7AAD –ve.

(C) Only the Annexin V/7AAD double-positive population from Supplementary Fig. 2 B was used to clarify the insignificance among reprogrammed MEFs uninfected or infected either by scrambled shRNA or two shRNAs targeting *Alkbh5*.N. S; Not significant.

(D) Cell proliferation was assessed by FACS measured by BrdU incorporation on day 3 of reprogramming using uninfected MEFs or infected MEFs with either EV or ALKBH5-HA.

(E) Quantification of the mean percentage of each of the populations G1, S and G2/M from FACS data shown in supplementary Fig. 3D. The mean percentage of each population was written as the mean ± S.D.

(F) Cell proliferation assay on day 4 of reprogramming using uninfected MEFs or infected MEFs with either EV or ALKBH5-HA.

(G) Expression of G1 cell cycle regulators as assessed by qPCR at day 3 of reprogramming in reprogrammed MEFs uninfected or infected either by scrambled shRNA or two shRNAs targeting *Alkbh5* was estimated at day 3 of reprogramming. The data were normalized to the housekeeping gene *Gapdh*.

(H) Immunoblot analysis of the protein levels of several cell cycle regulators in either homozygous *Alkbh5*$^{f/f}$ MEFs or reprogrammed homozygous *Alkbh5*$^{f/f}$ MEFs on day 3 with or without treatment with 1 µM 4-OH Tam to remove *Alkbh5*. A-TUB was used as a loading control.

(I) Cell proliferation assay of homozygous *Alkbh5*$^{f/f}$ MEFs with or without treatment with 1 µM 4-OH Tam to remove *Alkbh5* at different time points.

(J) Cell cycle analysis detected by PI staining and analyzed by FACS in uninfected MEFs or infected with scrambled shRNA or two different shRNAs targeting *Alkbh5*.

(K) Quantification of G1, S, and G2/M cell cycle phase data (Supplementary Fig. 3J).

(L) Immunoblot analysis of the protein levels of several cell cycle regulators in homozygous *Alkbh5*$^{f/f}$ MEFs with or without treatment with 1 µM 4-OH Tam to remove Alkbh5. A-TUB used as a loading control.

(M) (M) FACS analysis of retroviral infection efficiency in MEFs using pMXs-DsRed after 3 days.

(N) (N) Bright-field and fluorescent images of Plate-E transfected with pMXs-DsRed vector. Scale bar 200µm.

(O) (O) Bright-field and fluorescent images of MEFs infected with either retroviral pMXs-DsRed or piggyback-TO-OSKM-mCherry treated with Dox. Scale bar 200µm.

(P) FACS analysis of SSEA1- and DsRed-positive populations of reprogrammed MEFs on days 7 and 14 of reprogramming using WT and KO Alkbh5 MEFs or MEFs infected with ALKBH5-HA.

(Q) FACS analysis of transfection efficiency in MEFs using piggyback-TO-OSKM-mCherry treated with Dox after 3 days.

(R) FACS analysis of SSEA1- and mCherry-positive populations of reprogrammed MEFs on days 7 and 14 of reprogramming using WT and KO Alkbh5 MEFs or MEFs infected with ALKBH5-HA.

Journal of Cell Science • Supplementary information

(S) Immunoblot analysis of the protein levels of mesenchymal and epithelial markers on day 6 of reprogramming after infection with either scrambled shRNA or two different shRNAs targeting *Alkbh5*. A-TUB was used as a loading control.

(T) Left panel shows FACS analysis of E-Cad- and PDGFRβ-positive populations using *Alkbh5*$^{f/f}$ MEFs with or without 1 µM 4-OH-Tam on day 6 of reprogramming. Right panel shows the optimization of gating. MEFs and ESCs were used as negative and positive markers for MET transition, respectively.

(U) The left panel shows FACS analysis of E-Cad- and Thy1-positive populations using *Alkbh5*$^{f/f}$ MEFs with or without 1 µM 4-OH-Tam on day 6 of reprogramming. Right panel shows the optimization of gating. MEFs and ESCs were used as negative and positive markers for MET transition, respectively.

(V) The left panel shows FACS analysis of E-Cad- and SSEA1-positive populations on day 14 of reprogramming. Right panel shows the optimization of gating. ESCs were used as a positive control.

(W) FACS analysis of Δ-PE-Oct4-GFP- and E-Cad-positive populations on day 14 of reprogramming using uninfected MEFs or infected with either *SCR* or two shRNAs targeting *Alkbh5*.

(X) Immunoblot analysis of the protein levels of both mesenchymal and epithelial markers in either homozygous *Alkbh5*$^{f/f}$ MEFs or reprogrammed homozygous *Alkbh5*$^{f/f}$ MEFs on day 6 with or without treatment with 1 µM 4-OH Tam to remove *Alkbh5*. A-TUB used as a loading control.

(Y) Phase contrast images of tracking morphological changes during reprogramming. Reprogrammed MEFs uninfected or infected either by scrambled shRNA or two shRNAs targeting *Alkbh5* were estimated at days 2, 4, 6 and 14 of reprogramming. Scale bar 200µm. Data are shown as the mean ± SD; n = 3, *P < 0.05, **P < 0.01, and ***P < 0.001.

Journal of Cell Science • Supplementary information

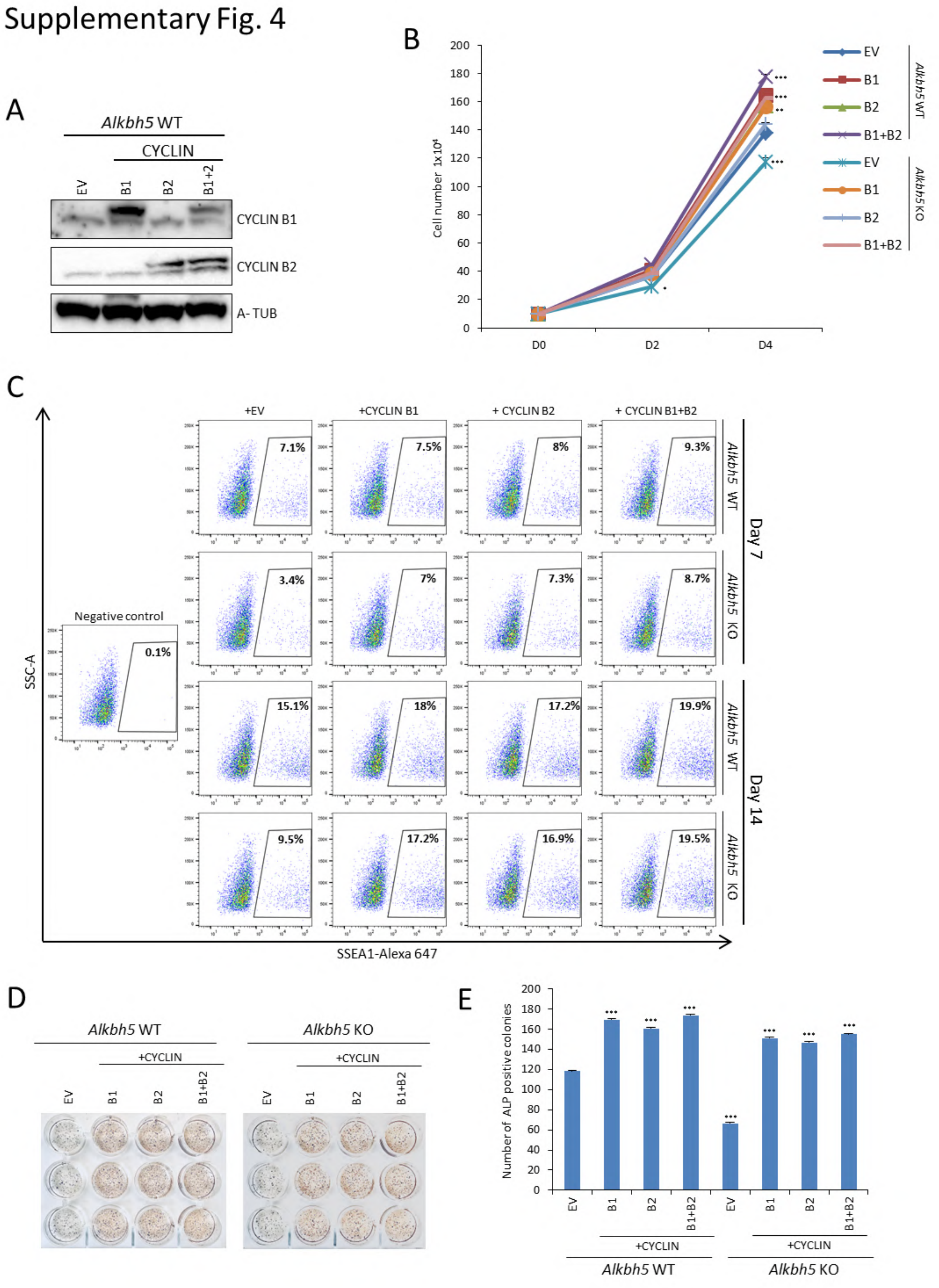

Fig. S4. CYCLIN B1 and/or B2 proliferation overexpression enhances both cell and reprogramming.

(A) Immunoblot of CYCLIN B1 and B2 in WT *Alkbh5*MEFs infected with either empty vector (EV), CYCLIN B1, B2, or both together. A-TUB used as a loading control.

(B) Cell proliferation assay on day 4 of reprogramming. Both WT and KO *Alkbh5* MEFs were infected with empty vector (EV), CYCLIN B1, B2, or both.

(C) Fraction of SSEA1-positive cells determined by FACS in reprogrammed MEFs on days 7 and 14 of reprogramming. Both WT and KO *Alkbh5* MEFs were infected with empty vector (EV), CYCLIN B1, B2, or both. Unstained MEFs used as a negative control.

(D) Representative image of ALP staining on day 14.

(F) Reprogramming efficiency was measured by counting the number of ALP-positive colonies represented in Supplementary Fig. 4D. Data are shown as the mean ± SD; n = 3, *P < 0.05, **P < 0.01, and ***P < 0.001 mean ± SD deviation of triplicate samples.

Journal of Cell Science • Supplementary information

Supplementary Fig. 5

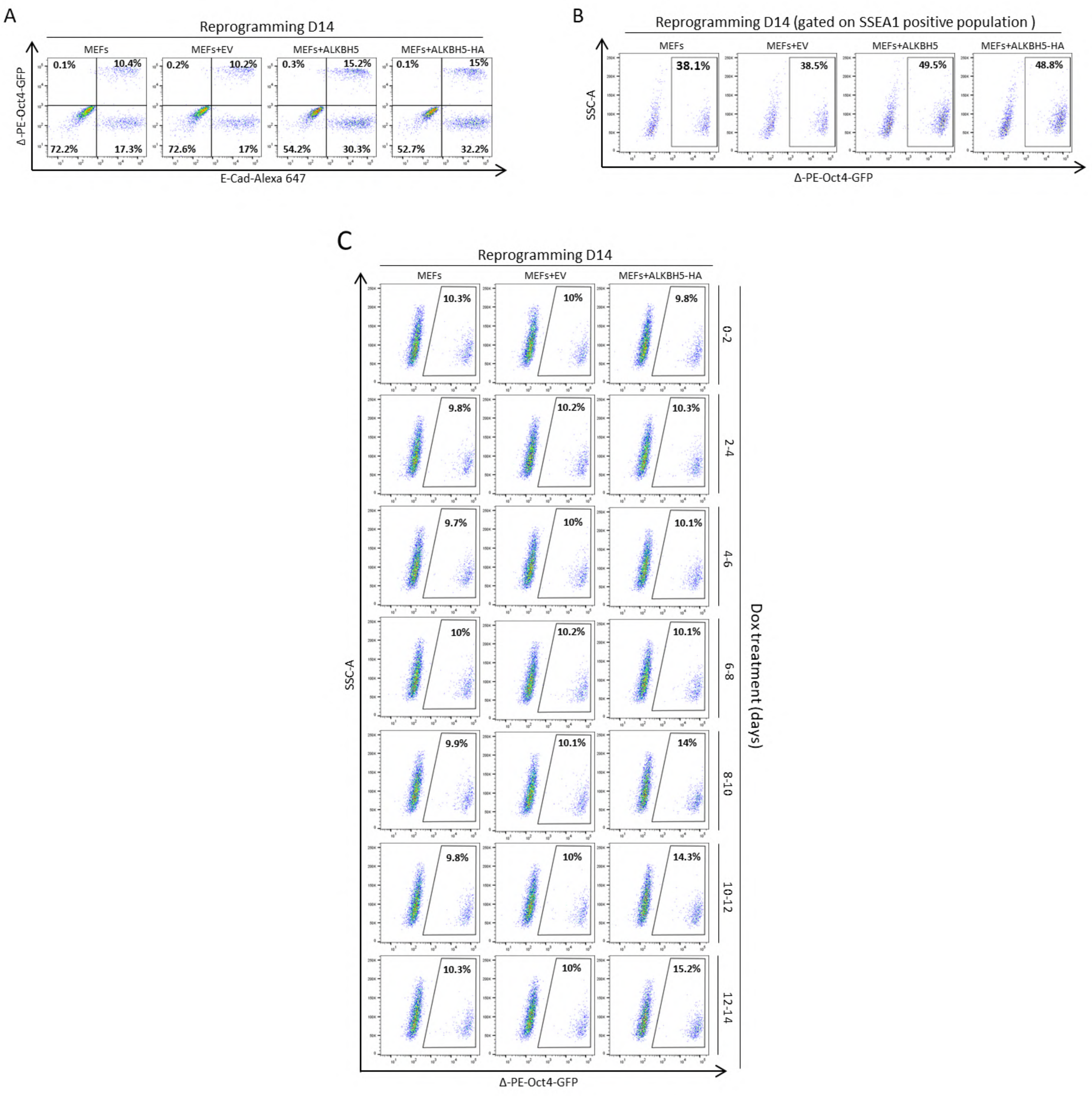

Fig. S5. ALKBH5 overexpression in the late phase enhances reprogramming efficiency.

(A) FACS analysis of Δ-PE-Oct4-GFP- and E-Cad-positive populations on day 14 of reprogramming using uninfected MEFs or infected with EV, ALKBH5-HA, and ALKBH5-HA.

(B) FACS analysis of the Δ-PE-Oct4-GFP-positive population gated on the SSEA1-positive fraction on day 14 of reprogramming.

(C) FACS analysis of the Δ-PE-Oct4-GFP-positive population throughout the whole reprogramming process. Uninfected MEFs and MEFs infected with EV or ALKBH5-HA were induced by Dox treatment every two days and analyzed on day 14.

Journal of Cell Science • Supplementary information

Supplementary Fig. 6

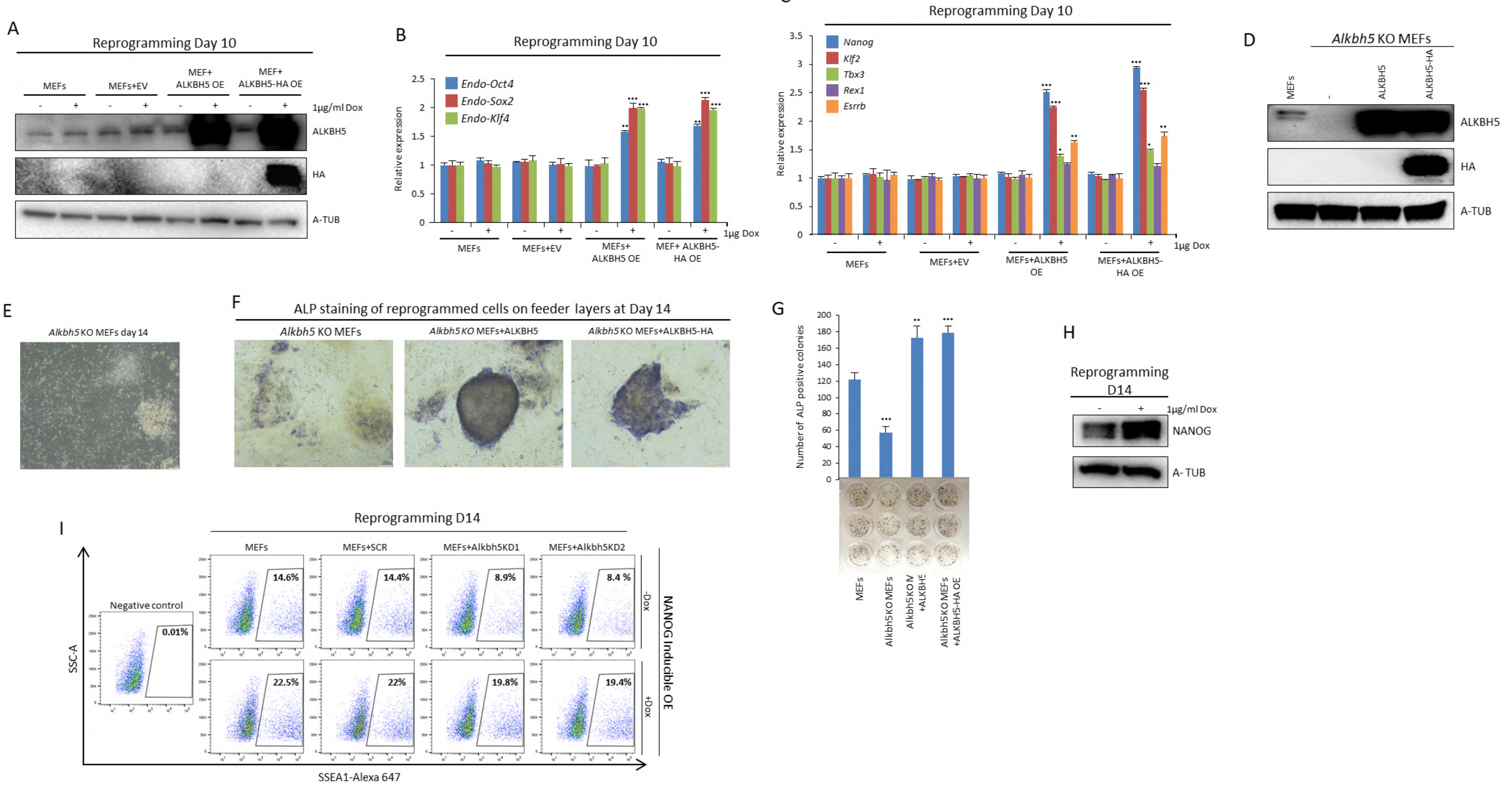

Fig. S6. ALKBH5 overexpression in the late phase of reprogramming enhances reprogramming efficiency by increasing *Nanog* expression.

(A) Immunoblot analysis of ALKBH5 protein levels after lentiviral infection of reprogrammed MEFs on day 12 with empty vector, ALKBH5 or ALKBH5-HA. Dox (1 µg/ml) was added on day 8, and the cells were harvested on day 10. A-TUB used as loading control.

(B) Endogenous expression of pluripotency factors (*Oct4*, *Sox2*, *Klf4*) as detected by qPCR on day 10 of reprogramming using either empty vector, ALKBH5 or ALKBH5-HA. The data were normalized to the housekeeping gene *Gapdh*.

(C) Expression of pluripotency markers detected by qPCR on day 12 of reprogramming using EV, ALKBH5 or ALKBH5-HA. The data were normalized to the housekeeping gene *Gapdh*.

(D) Immunoblot of ALKBH5 in WT and KO *Alkbh5* MEFs and rescued KO MEFs infected with lentiviral ALKBH5 and ALKBH5-HA. A-TUB used as a loading control.

(E) Phase contrast image of *Alkbh5* KO reprogrammed MEFs at day 14 of reprogramming. Scale bar 200µm.

(F) Phase contrast image of ALP-stained reprogrammed *Alkbh5* KO MEFs and rescued KO MEFs infected with either ALKBH5 or ALKBH5-HA at day 14. Scale bar 200µm.

(G) Reprogramming efficiency as assessed by counting the number of ALP-positive colonies on day 14 of reprogramming.

(H) Immunoblot of NANOG in MEFs infected with Dox-inducible NANOG with or without Dox treatment on day 14. A-TUB used as a loading control.

(I) Fraction of SSEA1-positive cells determined by FACS in reprogrammed MEFs on day 14 of reprogramming. NANOG was induced after 1 µg/ml Dox treatment. Data are shown as the mean ± SD; n = 3, *P < 0.05, **P < 0.01, and ***P < 0.001.

Journal of Cell Science • Supplementary information

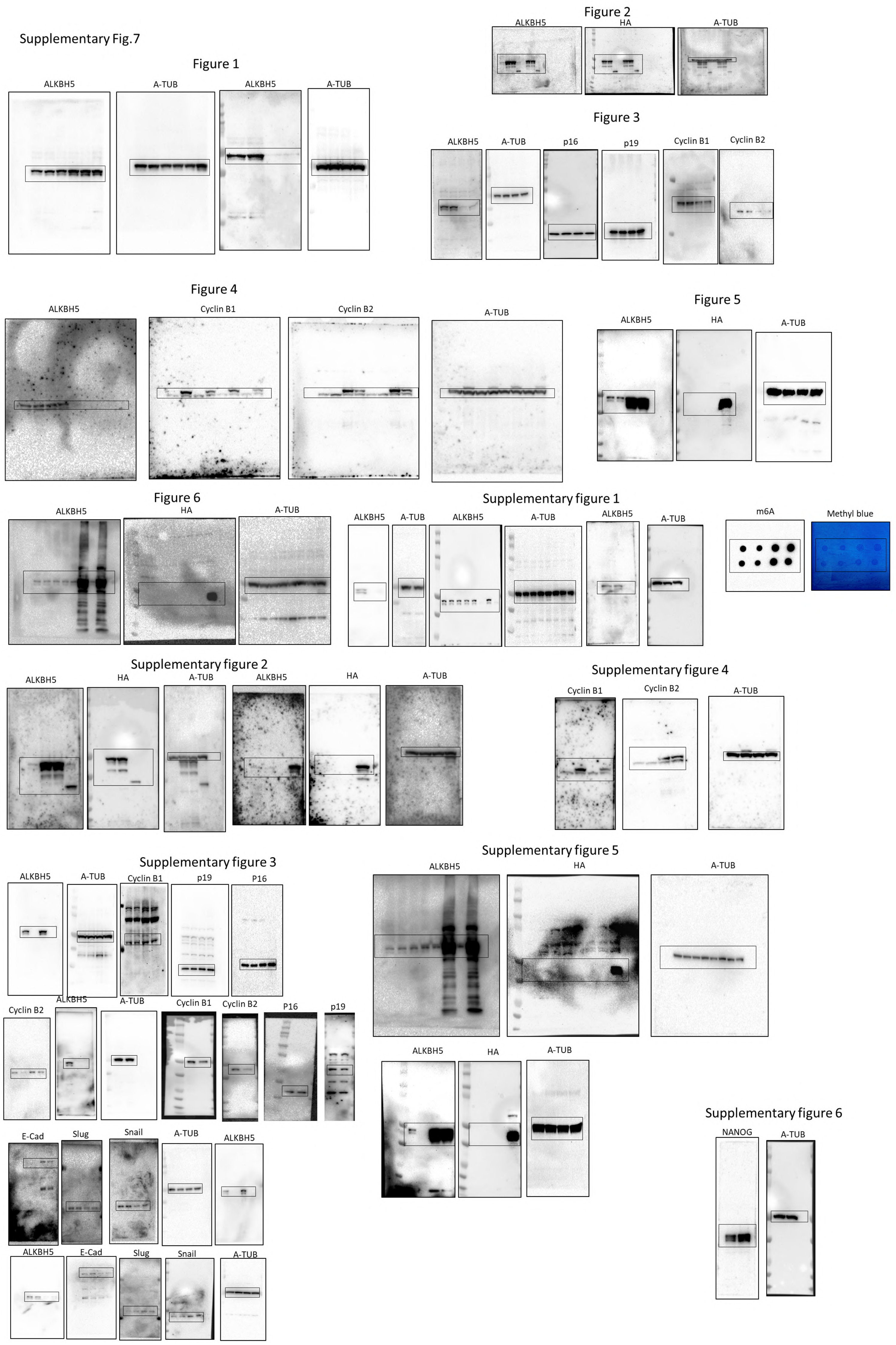

Fig. S7. Blot transparency for all western blot data including main and supplementary figures.

Journal of Cell Science • Supplementary information

Table S1. List of primers for qPCR, cloning and m6A IP

Click here to download Table S1

Table S2. List of antibodies used

Click here to download Table S2

Journal of Cell Science • Supplementary information
