# Supplementary material for: ALKBH5 regulates somatic cell reprogramming in a phase-specific manner
Source: J Cell Sci. 2022 Jun 10;135(11):jcs259824. doi: 10.1242/jcs.259824 (PMC9234673; doi:10.1242/jcs.259824)
Supplement: Reviewer comments [file joces-135-259824_review_history.pdf]

ALKBH5 regulates somatic cell reprogramming in a phase specific manner

Sherif Mahrous Khodeer, Arne Klungland and John Arne Dahl
DOI: 10.1242/jcs.259824

Editor: Caroline Hill

Review timeline

Submission to Review Commons:	11 May 2021
Submission to Journal of Cell Science:	26 January 2022
Editorial decision:	9 March 2022
First revision received:	5 April 2022
Accepted:	3 May 2022

Reviewer 1

Evidence, reproducibility and clarity

The authors show here that depletion of m6A demethylase Alkbh5 early during cellular reprogramming, impairs the reprogramming process. This effect is not observed when Alkbh5 is depleted in later stages of reprogramming. Furthermore, the authors show that KD of Alkbh5 brings to G2/M cell cycle arrest (apoptosis was not observed), and explain it by down-regulation of Cyclin B1&B2. In addition the authors show that MET is impaired as seen by decreasing the rate of upregulation of epithelial markers, and down-regulation of mesenchymal markers.
Finally, over-expression of ALKBH5 in the late stages of reprogramming enhances reprogramming efficiency by stabilizing the Nanog transcript.

The authors use a knock-down system by shRNA, as well as a conditional flox knock-out system to deplete Alkbh5, and prove an increase in m6A methylation. Reprogramming efficiency is estimated by SSEA1+ FACS sort and by AP staining. Levels of transcripts and proteins are measured by PCR and Western. Overall the results are clearly presented and the text is well written.

Major comment:

the authors selected several candidates to look at. In theory, the effect they describe may be mediated by other genes which were not measured. While I do not request that the authors add large-scale data (RNA-seq, m6a-seq) in this paper, they should at least discuss this option in their discussion part.

Minor comments:

Figure 1E: write "negative control" on the left panel (MEFs)

All bar plots: add the significance asterisk horizontally, this way it looks like data points. Indicate in the legend the type of statistical test used.

P8 line 9: add "and not due to cell apoptosis (Supplementary Fig. 2A-C)

P9 line 10: remove "by tracking" or rephrase this sentence

There is no Supp fig 3

© 2022. Published by The Company of Biologists under the terms of the Creative Commons Attribution License (https://creativecommons.org/licenses/by/4.0/).

Figure 4e: please change line types so it is possible to see MEFs Nanog line.

P11: 4th line 2nd paragraph: asbarriers

Significance

I see this paper not as a huge breakthrough, but rather as an additional part of the m6A puzzle, which was not described before, and fits well with previous evidence of the effect of m6a-manipulation in reprogramming (e.g. by depletion of Mettl3). I therefore think that the community that investigates m6A in the context of stem cells and development will benefit from the publication of this paper.

Reviewer 2

Evidence, reproducibility and clarity

This manuscript mainly uncovers the role of RNA m6A demethylase ALKBH5 in different phase of reprogramming. First, they find that depletion of Alkbh5 in the very early stage of reprogramming impairs the reprogramming process through down-regulating the expression of Cyclin B1 and B2, resulting in a reduction in the cell proliferation rate and mesenchymal- to-epithelial transition rate. Second, they discover that overexpression of Alkbh5 in the late stage enhances the reprogramming efficiency by stabilizing Nanog transcripts, leading to the up-regulation of Nanog expression. This work provides a new sight of how RNA m6A demethylase effects reprogramming. However, serval critical issues should be addressed and more information should be provided.

Comments and concerns:

Major points:

1. ALKBH5 is an RNA m6A demethylase. Whether the function of ALKBH5 in the reprogramming process depends on its enzymatic activities?
2. The in-depth mechanism of ALKBH5 in the reprogramming process is not fully explored. It is necessary to profile the target proteins of ALKBH5 in reprogramming. Whether the phenotype of ALKBH5 depletion can be rescued by the replenishment of its downstream targets.
3. At day 6 of reprogramming (Fig 2G), the mesenchymal and epithelial makers did not show significant changes after ALKBH5 knockdown, which make it hard to draw the conclusion that depletion of ALKBH5 impairs the MET process at protein level.

Minor points:

1. Gene was written incorrectly. For example, "Alkbh5" and "Nanog" should be italic.
2. Space are needed in several sentences. For example, "earlyreprogramming"(Abstract), "at day 6 of reprogramming in which Alkbh5was knocked down 2 days..."(Page 8).
3. Problems with verbs. Page 7, the first line, "suggests" should be "suggest"; Page 9, the second line, "impair" should be "impairs", and the last line, "is" should be "was"; Page 11, "the role of the m6A demethylases ALKBH5", "demethylases" should be "demethylase".
4. Problems with punctuation. Page 5, "including;" should be "including:". Page 14, the part of Reprogramming, "then the medium changes next day" should be "then the medium was changed next day"; In the part of Retrovirus preparation, "DMEM%10FBS" should be "DMEM+10%FBS". "4c" should be "4?". "1x106 cells per 10 cm dish" should be "1x106 cells per 10 cm dish". There are some same errors in the part of Lentivirus preparation.
5. Page 10, Mixed use of "Nanog transcripts" and "Nanog transcript", please write uniformly. Page 12, "DNA demethylating agents" should be "DNA demethylation agents".
6. Supplementary Fig 1I, the depletion of ALKBH5 seems not completely.
7. Supplementary Fig 2D, the deviation of the statistics is not acceptable.
8. Supplementary Fig 2K, the size of scale-bar should be elucidated clearly in the figure legend.
9. Supplementary Fig 4A, the blot of A-TUB is indistinct.
10. Supplementary Fig 4F, the statistic result of ALP staining should be showed.

© 2022. Published by The Company of Biologists under the terms of the Creative Commons Attribution License (https://creativecommons.org/licenses/by/4.0/).

11. Page 18, "1 to 2 μg of mRNA was fragmented for 4 minutes at 70? for 4 minutes", the first "for 4 minutes" should be deleted.
12. Page 19, "and then incubated with the primary antibody O.N was agitation", the "O.N was agitation" should be deleted.

Significance

average level to fit the journal.

Reviewer 3

Evidence, reproducibility and clarity

Summary:

The key findings of this paper are that the RNA m6A demethylase, ALKBH5 has different temporal effects on reprogramming of murine somatic cells to induced pluripotent stem cells. Using shRNA mediated knockdown or conditional knockout fibroblasts to deplete ALKBH5 they show that the depletion of ALKBH5 at the early stages of reprogramming (day 1-7) can reduce the generation of alkaline phosphatase (AP+) colonies. The overexpression of ALKBH5 at late stages of reprogramming (day 7-14) can increase the generation of AP+ colonies. They suggest that the reduction of reprogramming is due at least in part to alterations in cell cycle based on an increase in cells in G2/M and a slight reduction in overall cell proliferation due to the reduction of ALKBH5. The authors final conclusion is that this enhancement of reprogramming is through the stabilization of Nanog transcripts due to decreased m6a methylation resulting in the increased expression of the important pluripotency related protein.

Major comments:

- Are the key conclusions convincing?
The key conclusions are not convincing due to deficiencies in design of the experiments; modest effects on reprogramming efficiency and unsupported mechanism.

1) Alkaline phosphatase and SSEA1 are not bonafide reporters of iPSCs because even partially reprogrammed cells express these markers. Immunoflurescence for pluripotency markers such as Esrrb or Nanog would be required to make any conclusions.

2) Instead of just performing AP stains at the end of day 14, the SSEA+ cells are split onto plates. It is unclear why LIF is excluded from the reprogramming conditions for the first 7 days (Fig1D). This really confounds the temporal claim because the conditions on day 1 -7 are different and lacking a key pluripotent cell signaling through the LIF receptor, than on days 7-14.

3) The conclusions presented in figure 2 are underwhelming because the cell cycle effects on cyclin B1 do not translate to a large difference in cell number by day 4. More concerningly the effects on cell cycle also occur in non-reprogramming MEFs. Since reprogramming was induced with individual retroviruses, and the population was not selected for retroviral expression. So most of the cells in the population are likely to be untransduced MEFs. Therefore the claim that the effects of Alkbh knockdown on cell cycle are reprogramming specific is unsupported.

4) Similarly the claims of effects on MET by western blot are not quantified and seem weak.When Alkbh is over expressed after day 7 of reprogramming, whether MET or cell cycle are enhanced is not considered at all. The increase in E-cadherin and the reduction in Slug/ Snail is very small, in the Alkbh knockdown suggesting that neither MET nor transition to fast cycling like in ES cells is complete at day 7 even though the authors choose it as their "early" timepoint.

5) The conclusion that an increase in reprogramming efficiency after day 7 is a Nanog specific effect is unconvincing and premature. Figure 4D and 4E are problematic because GAPDH is being used as a control for the m6a methylation status of Nanog but this does not appear to be a m6a modified transcript (the m6a RIP for GAPDH is at input levels). The overexpression of ALKBH5 as

© 2022. Published by The Company of Biologists under the terms of the Creative Commons Attribution License (https://creativecommons.org/licenses/by/4.0/).

rescue serves a circular argument. It is known that the m6a modification leads to the degradation of transcripts and the general overexpression of ALKBH5 may lead to the increased stability of all m6a modified transcripts, not specifically Nanog or other pluripotency related factors. If the authors truly believe that the increase in AP positive colonies on Alkbh over expression is due to Nanog, then the knockout should be rescued with Nanog alone, not just Alkbh.

- Should the authors qualify some of their claims as preliminary or speculative, or remove them altogether?
Most of the conclusions are speculative. Better controlled true reprogramming (iPSCs) needs to be measured. Alternative explanations and experiments need to be thought through.
- Would additional experiments be essential to support the claims of the paper? Request additional experiments only where necessary for the paper as it is, and do not ask authors to open new lines of experimentation.
Evidence that other m6a modified transcripts are not maintained upon the overexpression of ALKBH5 would provide proof that this is a Nanog specific phenomenon. Unbiased RNA IP sequencing upon the overexpression of ALKBH5 could clarify what is happening. If total sequencing is not affordable, performing similar experiments as in Figure 4D and 4E on transcripts that are known to be m6a modified would at the very least show it is a Nanog specific effect in a small group, GAPDH does not appear to be the proper control in this situation.
Authors should check the effects on cell cycle when they overexpress ALKBH5 like they do for ALKBH5 reduction.
- Are the suggested experiments realistic in terms of time and resources? It would help if you could add an estimated cost and time investment for substantial experiments.
These types of experiments should not take more than 1-2 months depending on access to sequencing facilities.
- Are the data and the methods presented in such a way that they can be reproduced? Yes, the methodology appears to be thorough enough that these experiments could be replicated elsewhere.
- Are the experiments adequately replicated and statistical analysis adequate?
Experiments appear to be done with 3 independent replicates so we believe they have been adequately replicated.

Minor comments:

- Specific experimental issues that are easily addressable.
As mentioned above we are unsure why the authors are using abnormal reprogramming conditions such as no LIF for the first 7 days and less FBS. Changing this could potentially improve their reprogramming efficiency and they may see stronger effects when manipulating ALKBH5 expression. Some estimate of retroviral OSKM transduction efficiency is required.
The use of "MEFs" to indicate both unperturbed MEFs undergoing reprogramming as well as MEFs which do not have OSKM transduction like in Supp Fig 2 is confusion.
- Are prior studies referenced appropriately?
Aguillo et al. PMID: 26526723 is missing and is a highly relevant paper to this group's findings. They essentially do the inverse experiment of these authors by depleting Zfp217. Zfp217 sequesters the m6a methyltransferase METTL3 so the depletion of Zfp217 leads to global increases in m6a modified transcripts which results in the reduction of Nanog, Sox2, Klf4, c-Myc transcripts. Since this is known, some of the effects seen by the overexpression of ALKBH5 are expected.
- Are the text and figures clear and accurate?
There are a few minor typos in the text such as missed spaces between words but nothing too major. Figures are clear. Supplementary Figure 4 needs to be relabeled as Supp Fig 3.
- Do you have suggestions that would help the authors improve the presentation of their data and conclusions?
SCR is never defined in the text. Figure legend 1 mentions scrambled shRNA but the acronym is never defined.

Significance

- Describe the nature and significance of the advance (e.g. conceptual, technical, clinical) for the field.
The temporal requirements of factors changing their roles during reprogramming is well know for example Ho et.al. PMID: 23791530. In fact even the reprogramming factors themselves can be

© 2022. Published by The Company of Biologists under the terms of the Creative Commons Attribution License (https://creativecommons.org/licenses/by/4.0/).

inhibitory to the stabilization phase of reprorgamming PMID: 23217423 So the fact that Alkbh also can have differing temporal effects is not novel.
- Place the work in the context of the existing literature (provide references, where appropriate). This work is similar in nature to Aguillo 2015, which increased the global levels of m6a in ESCs and reprogramming cells and found this increased m6a inhibited somatic cell reprogramming. Khodeer et al is either decreasing global m6a levels or increasing them by manipulating ALKBH5 expression and sees effects on reprogramming that trend in the same directions as Aguillo et. al.
- State what audience might be interested in and influenced by the reported findings. As presented the findings are not convincing enough
- Define your field of expertise with a few keywords to help the authors contextualize your point of view. Indicate if there are any parts of the paper that you do not have sufficient expertise to evaluate.
Repogramming of somatic cells to iPSCs and epigenetic regulation.

Author response to reviewers' comments

We would like to thank the referees for their constructive comments, which we have used as the basis for revising and improving our manuscript.

Referees' Comments:

Reviewer #1 (Evidence, reproducibility and clarity (Required)):

The authors show here that depletion of m6A demethylase Alkbh5 early during cellular reprogramming impairs the reprogramming process. This effect is not observed when Alkbh5 is depleted in later stages of reprogramming. Furthermore, the authors show that KD of Alkbh5 brings to G2/M cell cycle arrest (apoptosis was not observed), and explain it by down-regulation of Cyclin B1&B2. In addition the authors show that MET is impaired as seen by decreasing the rate of upregulation of epithelial markers, and down-regulation of mesenchymal markers. Finally, over-expression of ALKBH5 in the late stages of reprogramming enhances reprogramming efficiency by stabilizing the Nanog transcript.

The authors use a knock-down system by shRNA, as well as a conditional flox knock-out system to deplete Alkbh5, and prove an increase in m6A methylation. Reprogramming efficiency is estimated by SSEA1+ FACS sort and by AP staining. Levels of transcripts and proteins are measured by PCR and Western. Overall the results are clearly presented and the text is well written.

Major comment:

The authors selected several candidates to look at. In theory, the effect they describe may be mediated by other genes which were not measured. While I do not request that the authors add large-scale data (RNA-seq, m6a-seq) in this paper, they should at least discuss this option in their discussion part.

Response: We Thank the Reviewer for this comment; we have now included a short paragraph on this at P18 Lines 24-25, and P19 Lines1-2.

Minor comments:

1) Figure 1E: write "negative control" on the left panel (MEFs).

Response 1: We have now corrected this in the revised manuscript. See Figure 1G of the revised manuscript.

2) All bar plots: add the significance asterisk horizontally, this way it looks like data points. Indicate in the legend the type of statistical test used.

© 2022. Published by The Company of Biologists under the terms of the Creative Commons Attribution License (https://creativecommons.org/licenses/by/4.0/).

Response 2: We have now added the significance asterisk horizontally in the revised version for all Figures.

3) P8 line 9: add "and not due to cell apoptosis (Supplementary Fig. 2A-C).

Response 3: We have now corrected this in the revised manuscript P10 Line 23.

4) P9 line 10: remove "by tracking" or rephrase this sentence.

Response 4: We have now edited this sentence in the revised version P13 Lines 17-19.

5) There is no Supp fig 3.

Response 5: We have now added two new Figures, and all main and supplementary Figures are adjusted accordingly.

6) Figure 4e: please change line types so it is possible to see MEFs Nanog line.

Response 6: We have now changed this in the revised manuscript and we have also added another negative control, *Stat3,* as shown in Figure 6 E.

7) P11: 4th line 2nd paragraph: as barriers

Response 7: We have now corrected this in the revised version P18 Line 11.

Reviewer #1 (Significance (Required) :

I see this paper not as a huge breakthrough, but rather as an additional part of the m6A puzzle, which was not described before, and fits well with previous evidence of the effect of m6a-manipulation in reprogramming (e.g. by depletion of Mettl3). I therefore think that the community that investigates m6A in the context of stem cells and development will benefit from the publication of this paper.

Response: We thank the reviewer for his evaluation and supportive comments.

Reviewer #2 (Evidence, reproducibility and clarity (Required)):

This manuscript mainly uncovers the role of RNA m6A demethylase ALKBH5 in different phase of reprogramming. First, they find that depletion of Alkbh5 in the very early stage of reprogramming impairs the reprogramming process through down-regulating the expression of Cyclin B1 and B2, resulting in a reduction in the cell proliferation rate and mesenchymal-to-epithelial transition rate. Second, they discover that overexpression of Alkbh5 in the late stage enhances the reprogramming efficiency by stabilizing Nanog transcripts, leading to the up-regulation of Nanog expression. This work provides a new sight of how RNA m6A demethylase effects reprogramming. However, several critical issues should be addressed and more information should be provided.

Comments and concerns:

Major points:

- ALKBH5 is an RNA m6A demethylase. Whether the function of ALKBH5 in the reprogramming process depends on its enzymatic activities?

Response 1: We thank the reviewer for his helpful comment; we have now addressed this point and the data is presented in main Figure 2 and supplementary Figure 2 of the revised manuscript. For this, we have constructed catalytically inactive ALKBH5-HA (H205A), and catalytically deleted ALKBH5-HA (CD). The data revealed that ALKBH5 regulates the somatic reprogramming

© 2022. Published by The Company of Biologists under the terms of the Creative Commons Attribution License (https://creativecommons.org/licenses/by/4.0/).

through its catalytic activity.

- The in-depth mechanism of ALKBH5 in the reprogramming process is not fully explored. It is necessary to profile the target proteins of ALKBH5 in reprogramming. Whether the phenotype of ALKBH5 depletion can be rescued by the replenishment of its downstream targets.

Response 2: We thank the reviewer for the comment. Our preliminary unpublished data regarding the role of ALKBH5 in pluripotency revealed that *Cyclin B1* and *Cyclin B2* are m6A regulated transcripts. We have now addressed this point in main Figure 4 and supplementary Figure 4 of the revised version. Our m6A-qPCR data revealed that both *Cyclin B1* and *B2 transcripts* are more enriched with m6A in *Alkbh5* KO MEFs compare to WT on day 3 of reprogramming (Figure 4E), which in turn results in decreasing their stability (Figure 4D). Besides overexpression CYCLIN B1 AND B2 alone or both together can rescue the phenotype of *Alkbh5* depletion in somatic reprogramming (Figure 4A-C and Supplementary Figure 4C-E).

- At day 6 of reprogramming (Fig 2G), the mesenchymal and epithelial makers did not show significant changes after ALKBH5 knockdown, which make it hard to draw the conclusion that depletion of ALKBH5 impairs the MET process at protein level.

Response 3: We thank the reviewer for this observation. Indeed on day 6 of reprogramming we observed that the level of mesenchymal transcripts in *Alkbh5* knockdown MEFs were not downregulated as seen in WT MEFs. While on the other hand, the levels of epithelial transcripts in *Alkbh5* knockdown were not upregulated as in WT MEFs (Figure. 3F of the revised version). On the protein level, our immunoblot methodology was not sensitive enough for quantifying the change at the protein level (Supplementary Figure 3S and T of the revised version). To address the point raised by the reviewer, we used flow cytometry for precise estimation of the change on both mesenchymal and epithelial markers at the protein level. Our data revealed that *Alkbh5* knockout MEFs have impaired upregulation of the epithelial marker E-Cadherin as compared to WT MEFs, but we did not observe any significant change on mesenchymal markers PDGFRB and Thy1 (Figure 3G-I and supplementary Figure 3U-X). We have now described these findings in the revised manuscript at P3 Lines 11-12, P12 Lines 9-25, P13 Lines 1-20, and P19 Lines 5- 8.

<u>**Minor points:**</u>

1. Gene was written incorrectly. For example, "Alkbh5" and "Nanog" should be italic.

Response 1: We have now corrected all gene names accordingly in the revised version.

2. Spaces are needed in several sentences. For example, "earlyreprogramming"(Abstract), "at day 6 of reprogramming in which Alkbh5was knocked down 2 days..."(Page 8).

Response 2: We have now thoroughly checked and corrected all spaces and formatting. The mentioned examples are corrected in the revised version, P3 Line 14 and P6 Lines 14-15.

3. Problems with verbs. Page 7, the first line, "suggests" should be "suggest"; Page 9, the second line, "impair" should be "impairs", and the last line, "is" should be "was"; Page 11, "the role of the m6A demethylases ALKBH5", "demethylases" should be "demethylase".

Response 3: We have now corrected all grammar in the revised version. P7 Line 14 “suggest”, P12 Line 10 “impairs”, P16 Line 2 “was”, P18 Line 2 “demethylase”.

4. Problems with punctuation. Page 5, "including;" should be "including:". Page 14, the part of Reprogramming, "then the medium changes next day" should be "then the medium was changed next day"; In the part of Retrovirus preparation, "DMEM%10FBS" should be "DMEM+10%FBS". "4c" should be "4℃". "1x106 cells per 10 cm dish" should be "1x106 cells per 10 cm dish". There are some same errors in the part of Lentivirus preparation.

Response 4: We have now corrected these issues in the revised version. P5 Line 18 “including:” P25 Line 3 “then the medium was changed next day”, P26 Line 3 “DMEM+10%FBS”, "4℃" was

© 2022. Published by The Company of Biologists under the terms of the Creative Commons Attribution License (https://creativecommons.org/licenses/by/4.0/).

corrected in all material and methods part, 1x10^6 and other superscripts were corrected in materials and methods part. We have also rechecked the lentivirus preparation part P26 Line 13-23.

5. Page 10, mixed use of "Nanog transcripts" and "Nanog transcript", please write uniformly. Page 12, "DNA demethylating agents" should be "DNA demethylation agents".

Response 5: We have now uniformly used *Nanog* transcripts throughout the manuscript. Corrected at P3 Line 16, P6 Line 4, P16 Lines 20, 23 and 25, P17 Lines 1-2, Lines 15-16, and P19 Lines 21-22. We have also corrected “DNA demethylation agents” P19 Line 17.

6. Supplementary Fig 1I, the depletion of ALKBH5 seems not completely.

Response 6: Thank you for noticing this. We have now replaced 8 hour 4-OH-Tam treatment of *Alkbh5*$^{f/f}$ with 24 hour treatment which showed the complete depletion of *Alkbh5* in the revised version (Supplementary Figure 1L).

7. Supplementary Fig 2D, the deviation of the statistics is not acceptable.

Response 7: We have now replaced this with newly generated data (Supplementary Figure 3G in the revised version).

8. Supplementary Fig 2K, the size of scale-bar should be elucidated clearly in the figure legend.

Response 8: We have now provided this information in the legend of Supplementary Figure 3Y in the revised version.

9. Supplementary Fig 4A, the blot of A-TUB is indistinct.

Response 9: We have now replaced that with new data (Supplementary Figure 6A in the revised version).

10. Supplementary Fig 4F, the statistic result of ALP staining should be showed.

Response 10: We are now showing this in the Supplementary Figure 6G in the revised version.

11. Page 18, "1 to 2 µg of mRNA was fragmented for 4 minutes at 70℃ for 4 minutes", the first "for 4 minutes" should be deleted.

Response 11: We have now deleted that, P30 Line 22.

12. Page 19, "and then incubated with the primary antibody O.N was agitation", the "O.N was agitation" should be deleted.

Response 12: We have now corrected this, P 31 Lines19-20.

Reviewer #2 (Significance (Required)):

Average level to fit the journal.

Response : We thank the reviewer for valuable comments and evaluation.

© 2022. Published by The Company of Biologists under the terms of the Creative Commons Attribution License (https://creativecommons.org/licenses/by/4.0/).

Reviewer #3 (Evidence, reproducibility and clarity (Required)):

Summary:

The key findings of this paper are that the RNA m6A demethylase, ALKBH5 has different temporal effects on reprogramming of murine somatic cells to induced pluripotent stem cells. Using shRNA mediated knockdown or conditional knockout fibroblasts to deplete ALKBH5 they show that the depletion of ALKBH5 at the early stages of reprogramming (day 1-7) can reduce the generation of alkaline phosphatase (AP+) colonies. The overexpression of ALKBH5 at late stages of reprogramming (day 7-14) can increase the generation of AP+ colonies. They suggest that the reduction of reprogramming is due at least in part to alterations in cell cycle based on an increase in cells in G2/M and a slight reduction in overall cell proliferation due to the reduction of ALKBH5. The author's final conclusion is that this enhancement of reprogramming is through the stabilization of Nanog transcripts due to decreased m6a methylation resulting in the increased expression of the important pluripotency related protein.

Major comments:

- Are the key conclusions convincing?

The key conclusions are not convincing due to deficiencies in design of the experiments; modest effects on reprogramming efficiency and unsupported mechanism.

1. Alkaline phosphatase and SSEA1 are not bonafide reporters of iPSCs because even partially reprogrammed cells express these markers. Immunoflurescence for pluripotency markers such as Esrrb or Nanog would be required to make any conclusions.

Response 1: We agree with the reviewer in this point on that alkaline phosphatase staining and SSEA1 expression are not definitive markers for iPS reprogramming, but indeed the two suggested markers ("Esrrb or Nanog") are arguably not suitable for our study. Using either NANOG or its downstream target ESSRB, will create a biased estimation for the reprogramming efficiency, besides NANOG itself is dispensable for generation of iPSCs, and *Nanog* null iPSCs contribute to both tertoma and chimera [1]. Based on that, we decided to use transgenic MEFs (OG2) with Δ-PE-Oct4-GFP reporter as a definitive and the most stringent marker for estimation of the reprograming efficiency, naïve pluripotency and primordial germ cell (PGC) specification [2-4]. We received the transgenic MEFs (OG2) with Δ-PE-Oct4-GFP reporter as a kind gift from professor Hans R. Schöler (Department of Cell and Developmental Biology, Max Planck Institute for Molecular) [5]. We have put major efforts into getting this right, and we have now repeated all our relevant experiments using this reporter (transgenic MEFs (OG2) with Δ-PE- Oct4-GFP reporter). Please kindly see the revised version (Figure 1D-F, Figure 2C and D, Figure 4B and C, Figure 5B and C, Figure 6F and G, Supplementary Figure 1I, Supplementary Figure 3X, and Supplementary Figure 5A-C.

2. Instead of just performing AP stains at the end of day 14, the SSEA+ cells are split onto plates. It is unclear why LIF is excluded from the reprogramming conditions for the first 7 days (Fig1D). This really confounds the temporal claim because the conditions on day 1 -7 are different and lacking a key pluripotent cell signaling through the LIF receptor, than on days 7-14.

Response 2: We thank the reviewer for raising this point so that we can clearify this. We have intentionally excluded LIF from the early phase as to keep our reprogramming protocol very similar to the basic reprogramming protocol from the Yamanaka group that is also using the same retroviral system (pMX-s), in which Leukemia inhibitiory factor (LIF) is added in the late stage of reprogramming, according to as described and validated in their publication (Figure 4, Panel A) [6]. Indeed our optimized and published protocol [7] is in agreement with other findings indicating that LIF is required in the late phase of reprogramming to promote converting pre-iPS cells or partially reprogrammed iPS cells to fully reprogrammed iPS cells through epigenetic regulation mediated by facilitating DNA demethylation/de novo methylation [8]. Furthermore, LIF and its downstream mediators Janus kinase/signal transducer and activator of transcription 3 (Jak/Stat3) signaling pathway upregulate its downstream target Esrrb in the late phase to ensure

© 2022. Published by The Company of Biologists under the terms of the Creative Commons Attribution License (https://creativecommons.org/licenses/by/4.0/).

complete reproframming [9]. Moreover, Jak/Stat3 signalling patwhay has been reproted to be essentail in the late phase of reprogramming by enhancing the demehtylation of pluripotency loci Oct4, Nanog, and genomic imprinting locus Dlk1-Dio3 whithout shoing any effect on MET at the early phase of reprogramming [10]. In addition to this extensive publihsed support, we also provide experimental evidence to address the point raised by the reviewer. We have now generated data on reprogramming including LIF treatment either from day 0 to day 7, or day 7 to day 14, or from day 0 to day 14, which clealry indicate that LIF is required in the late phase of reprogramming as shown by an increased fraction of Δ-PE-Oct4-GFP positive cells, used as a definitive marker for reprogramming. The data is provided as an attachment to this response to the reviewers. **(Attached file named "Reply to reviewer 3 major comment No.2")**

Regarding assessment of SSEA1, we would like to clarify that for every design we run two experiments in parallel. In the first one, we trypsinize the cells at day 7 and do both the measuring of the SSEA1 positive cells by FACS, and the plating of 5000 cells on feeder layers and then ALP staining at day 14. While in the second one, we let the cells grow till day 14, then we trypsinize them and measure the SSEA1 positive fractions. It is practially challenging to do ALP directly on day 14 without spiliting the cells, as the colonies would then grow into each other to become merged and it would not be feasible to accuretly count and provide a proper and accurate estimation of reprogramming efficiency. Therefore we argue that our set up provide more replicates and also ensure proper assessment of the reprogramming efficiency.

3. The conclusions presented in figure 2 are underwhelming because the cell cycle effects on cyclin B1 do not translate to a large difference in cell number by day 4. More concerningly the effects on cell cycle also occur in non-reprogramming MEFs. Since reprogramming was induced with individual retroviruses, and the population was not selected for retroviral expression. So most of the cells in the population are likely to be untransduced MEFs. Therefore the claim that the effects of Alkbh knockdown on cell cycle are reprogramming specific is unsupported.

Response 3: We thank the reviewer for this comment. Our statistical analysis revealed that there is a significant difference in cell number and the cell cycle during the reprogramming (Figure 3A-C in the revised manuscript). Nevertheless, we in general understand the reviewers concern about reprogramming retroviral vectors (pMX-s Oct4, Sox2, Klf4, and c-Myc) lacking selective markers. However, to overcome such issues we have used a control vector pMX-s DsRED (Addgene #22724) to monitor the infection efficiency in accordance with what has been reported in the Yamanka protocol [11]. Our results revealed that the infection efficiency is exceeds 90% as accurately measured by FACS, and the SSEA1 positive population emerges from the DsRED positive population (revised version Supplementary figure 3M-P). Furthermore, we used the polycistronic piggyback system PB-TAC-OSKM (Addgene #80481) in which m- Cherry is downstream of IRES to precisely monitor the reprogrammed population. In consistence with our retroviral data, we observed that the SSEA1 positive population emerges from the m-Cherry positive population (revised version supplementary figure 3O, Q and R). All together, these data suggested that not only is the impact of *Alkbh5* reprogramming specific, but the data are also consistent for both reprogramming methodologies, as we now have discussed in the revised manuscript P11 Lines 14-25 and P12 Lines 1-7.

4. Similarly the claims of effects on MET by western blot are not quantified and seem weak.When Alkbh is over expressed after day 7 of reprogramming, whether MET or cell cycle are enhanced is not considered at all. The increase in E-cadherin and the reduction in Slug/ Snail is very small, in the Alkbh knockdown suggesting that neither MET nor transition to fast cycling like in ES cells is complete at day 7 even though the authors choose it as their "early" timepoint.

Response 4: We appreciate the reviewer comment and this observation. Indeed, we observed that on day 6 of reprogramming the levels of mesenchymal transcripts in *Alkbh5* knockdown MEFs are not downregulated as they are in WT MEFs. While the levels of epithelial transcripts in *Alkbh5* knockdown were not upregulated as they are in WT MEFs (Figure. 3F of the revised manuscript). On the protein level, our immunoblot methodology was not sensitive enough for quantifying the change at the protein level (Supplementary Figure 3S-T of the revised manuscript). To overcome this, we have now used flow cytometry for precise estimation of the

© 2022. Published by The Company of Biologists under the terms of the Creative Commons Attribution License (https://creativecommons.org/licenses/by/4.0/).

change on both mesenchymal and epithelial markers at the protein level. Our data revealed that *Alkbh5* knockout MEFs show impaired upregulation of the epithelial marker E-Cadherin as compared to WT MEFs. Indeed we did not see any significant change at day 6 for mesenchymal markers PDGFRß and Thy1 as shown in (Figure. 3 G-I and supplementary Figure 3U-X). We have now clearly stated this in the revised manuscript at P3 Lines 11-12, P12 Lines 9-25, P13 Lines 1-20, and P19 Lines 5-8.

5. The conclusion that an increase in reprogramming efficiency after day 7 is a Nanog specific effect is unconvincing and premature. Figure 4D and 4E are problematic because GAPDH is being used as a control for the m6a methylation status of Nanog but this does not appear to be a m6a modified transcript (the m6a RIP for GAPDH is at input levels). The overexpression of ALKBH5 as rescue serves a circular argument. It is known that the m6a modification leads to the degradation of transcripts and the general overexpression of ALKBH5 may lead to the increased stability of all m6a modified transcripts, not specifically Nanog or other pluripotency related factors. If the authors truly believe that the increase in AP positive colonies on Alkbh over expression is due to Nanog, then the knockout should be rescued with Nanog alone, not just Alkbh.

Response 5: Indeed, we have reported in our previous work, that *Gapdh* is not an m6A modified transcript, and *Gapdh* is known as a stable transcript, that's why we have been using it as a negative control either in m6A-qPCR or in half-life time experiments [12]. In addition, we have now included *Stat3* as another negative control, which has been reported and used as a negative control for m6A in pluripotency in a previous publication [13]. The use of *Stat3* as a negative control confirmed our m6A-qPCR and half-life time findings as is now shown in the revised manuscript (Figure 6D and E).
Furthermore, to assess whether NANOG overexpression can rescue the *Alkbh5* KO reprogramming phenotype, we used a dox-inducible system for overexpression of NANOG in the late stage of reprogramming. Our data showed that NANOG overexpression can rescue the reprogramming efficiency in *Alkbh5* KO MEFs, and enhance the reprogramming efficiency in WT MEFs, as now reported in the revised manuscript (Figure 6 F and G, and Supplementary Figure 6 H and I). These results are now described at P17 Lines 6-13 of the revised manuscript.

- Should the authors qualify some of their claims as preliminary or speculative, or remove them altogether?

Most of the conclusions are speculative. Better controlled true reprogramming (iPSCs) needs to be measured. Alternative explanations and experiments need to be thought through.

Response: We have now repeated all relevant experiments using a definitive and stringent reporter (Δ-PE-Oct4-GFP) for precise evaluation of reprogramming efficiency by FACS coupled with both ALP staining and SSEA1 assessment, as described above under response 1.

- Would additional experiments be essential to support the claims of the paper? Request additional experiments only where necessary for the paper as it is, and do not ask authors to open new lines of experimentation.

Evidence that other m6a modified transcripts are not maintained upon the overexpression of ALKBH5 would provide proof that this is a Nanog specific phenomenon. Unbiased RNA IP sequencing upon the overexpression of ALKBH5 could clarify what is happening. If total sequencing is not affordable, performing similar experiments as in Figure 4D and 4E on transcripts that are known to be m6a modified would at the very least show it is a Nanog specific effect in a small group, GAPDH does not appear to be the proper control in this situation.

Response: We are thankful to the reviewer, however we would like to emphasize that we do not claim that this is a "Nanog specific phenomenon". Indeed, based on the literature it is expected that several pluripotency markers are regulated through the m6A modification [13-15]. Based on several published papers (as well as our unpublished m6A-seq from mESCs), we would expect that Nanog is a proper candidate as it is upstream of Esrrb [16], share lots of targets with OCT4 and

© 2022. Published by The Company of Biologists under the terms of the Creative Commons Attribution License (https://creativecommons.org/licenses/by/4.0/).

SOX2 [17, 18], and overlap with PRDM14 [19]. In addition to that, Nanog sustain the expression of *Oct4, Sox2, Esrrb, Rif1 FoxD3, Tcfcp2l1, Sall1, REST, Jarid2, Tcf3 and Nr0b1*[17]. Furthermore, Nanog KO ESC in serum/LIF has a severe phenotype compared to KO of other pluripotency markers, placing it next after Oct4 in the safeguarding of pluripotency. However, although published work provides support to our strategy, we now also provide experimental evidence to address the point raised by the reviewer. We have now included in the revised manuscript data on NANOG overexpression at the late stage of reprogramming that support our hypothesis regarding recapitulating the ALKBH5 overexpression. Further support is gained from our data showing enhanced reprogramming efficiency in WT MEFs overexpressing NANOG. Please see the revised manuscript (Figure 6 F and G, and supplementary Figure 6 H and I). This is now described and discussed at P17 Lines 6-13.

- Authors should check the effects on cell cycle when they overexpress ALKBH5 like they do for ALKBH5 reduction.

Response: We have now included data for cell cycle analysis of ALKBH5 overexpression on day 3 of reprogramming as shown in the revised manuscript (Supplementary Figure 3D-F). Our data support our hypothesis that ALKBH5 overexpression does not have any impact on cell cycle. It has been stated in the revised manuscript P10 Line 23-24 and P11 Line 1.

- Are the suggested experiments realistic in terms of time and resources? It would help if you could add an estimated cost and time investment for substantial experiments.
These types of experiments should not take more than 1-2 months depending on access to sequencing facilities.
- Are the data and the methods presented in such a way that they can be reproduced?

Yes, the methodology appears to be thorough enough that these experiments could be replicated elsewhere.
- Are the experiments adequately replicated and statistical analysis adequate?

Experiments appear to be done with 3 independent replicates so we believe they have been adequately replicated.

Minor comments:

- Specific experimental issues that are easily addressable.

As mentioned above we are unsure why the authors are using abnormal reprogramming conditions such as no LIF for the first 7 days and less FBS. Changing this could potentially improve their reprogramming efficiency and they may see stronger effects when manipulating ALKBH5 expression.

Response: We have replied to the point of using LIF in late stage of reprogramming above. Please see Response number 2. Regarding using low FBS, we intentionally used 10% FBS in our reprogramming procedure rather than serum free medium with N2B27 and two inhibitors for MAPK and GSK3 (2i/L). The rational is to avoid any influence from cell signaling cross-talk which might interfere with the results and interpretation. Several naïve pluripotency genes are down-stream of MAPK including NANOG, and its downstream target ESRRB, and PRDM14 [16, 20, 21]. In addition to that, NANOG itself has showed higher stability and uniform expression at the protein level after inhibition of MAPK as NANOG is a direct target for ERK1 which phosphorylates and trigger NANOG degradation through ubiquitination [22, 23]. Furthermore, the 10% of FBS is the minimum concentration required for proper cell proliferation. Decreasing the serum concentration further would decrease the reprogramming efficiency due to cell cycle arrest. Finally, achieving the 7% to 8% and 14% to 16% reprogramming efficiency at day 7 and day 14, respectively, are in the high end of the reported reprogramming efficiency using our reprogramming system. In conclusion, we would argue that our set-up and design fits well the points we aim to address.

© 2022. Published by The Company of Biologists under the terms of the Creative Commons Attribution License (https://creativecommons.org/licenses/by/4.0/).

Some estimate of retroviral OSKM transduction efficiency is required.

Response: We thank the reviewer for his comment. We have replied to this point above and include robust data. Please see Response number 3.

The use of "MEFs" to indicate both unperturbed MEFs undergoing reprogramming as well as MEFs which do not have OSKM transduction like in Supp Fig 2 is confusion.

Response: We are thankful to the reviewer for pointing out that this needed to be presented more clearly. We have now clearly stated in the revised manuscript text, figure legend of Figure 3 and in the figure itself where the MEFs are reprogrammed MEFs with the description “MEFs+OSKM”. Also we are using the description “reprogramming day 3” on the top of the figure to make this clear. While in Supplementary figure 3I-K, and the related figure legends we use the description “MEFs”. We believe that we now make a clear distinction in the revised manuscript.

- Are prior studies referenced appropriately?

Aguillo et al. PMID: 26526723 is missing and is a highly relevant paper to this group's findings. They essentially do the inverse experiment of these authors by depleting Zfp217. Zfp217 sequesters the m6a methyltransferase METTL3 so the depletion of Zfp217 leads to global increases in m6a modified transcripts which results in the reduction of Nanog, Sox2, Klf4, c-Myc transcripts. Since this is known, some of the effects seen by the overexpression of ALKBH5 are expected.

Response: We have now cited this reference in our revised manuscript at P19 Line 11.

- Are the text and figures clear and accurate?

There are a few minor typos in the text such as missed spaces between words but nothing too major. Figures are clear. Supplementary Figure 4 needs to be relabeled as Supp Fig 3.

Response: We have now thoroughly edited and corrected the mistyping etc in the revised manuscript, and we have labeled all figures properly.

- Do you have suggestions that would help the authors improve the presentation of their data and conclusions?

SCR is never defined in the text. Figure legend 1 mentions scrambled shRNA but the acronym is never defined.

Response: We have now clarified these things in the Figure 1C legends.

Reviewer #3 (Significance (Required)):

- Describe the nature and significance of the advance (e.g. conceptual, technical, clinical) for the field.

The temporal requirements of factors changing their roles during reprogramming is well know for example Ho et.al. PMID: 23791530. In fact even the reprogramming factors themselves can be inhibitory to the stabilization phase of reprorgamming PMID: 23217423 So the fact that Alkbh also can have differing temporal effects is not novel.

- Place the work in the context of the existing literature (provide references, where appropriate).

This work is similar in nature to Aguillo 2015, which increased the global levels of m6a in ESCs and reprogramming cells and found this increased m6a inhibited somatic cell reprogramming. Khodeer et al is either decreasing global m6a levels or increasing them by manipulating ALKBH5 expression and sees effects on reprogramming that trend in the same

© 2022. Published by The Company of Biologists under the terms of the Creative Commons Attribution License (https://creativecommons.org/licenses/by/4.0/).

directions as Aguillo et. al.

- State what audience might be interested in and influenced by the reported findings.

As presented the findings are not convincing enough

- Define your field of expertise with a few keywords to help the authors contextualize your point of view. Indicate if there are any parts of the paper that you do not have sufficient expertise to evaluate.

Repogramming of somatic cells to iPSCs and epigenetic regulation.

References

1. Schwarz, B.A., et al., *Nanog is dispensable for the generation of induced pluripotent stem cells.* Current Biology, 2014. **24**(3): p. 347-350.
2. Gafni, O., et al., *Derivation of novel human ground state naive pluripotent stem cells.*
Nature, 2013. **504**(7479): p. 282-286.
3. Murakami, K., et al., *NANOG alone induces germ cells in primed epiblast in vitro by activation of enhancers.* Nature, 2016. **529**(7586): p. 403.
4. Malik, V., et al., *Pluripotency reprogramming by competent and incompetent POU factors uncovers temporal dependency for Oct4 and Sox2.* Nature communications, 2019. **10**(1): p. 1-16.
5. Velychko, S., et al., *Excluding Oct4 from Yamanaka cocktail unleashes the developmental potential of iPSCs.* Cell Stem Cell, 2019. **25**(6): p. 737-753. e4.
6. Hong, H., et al., *Suppression of induced pluripotent stem cell generation by the p53–p21 pathway.* Nature, 2009. **460**(7259): p. 1132-1135.
7. Khodeer, S. and T. Era, *Identifying the biphasic role of calcineurin/NFAT signaling enables replacement of Sox2 in somatic cell reprogramming.* Stem cells, 2017. **35**(5): p. 1162-1175.
8. Tang, Y., et al., *Jak/Stat3 signaling promotes somatic cell reprogramming by epigenetic regulation.* Stem cells, 2012. **30**(12): p. 2645-2656.
9. Huang, D., et al., *LIF-activated Jak signaling determines Esrrb expression during late-stage reprogramming.* Biology open, 2018. **7**(1): p. bio029264.
10. Wang, L., et al., *JAK/STAT3 regulated global gene expression dynamics during late-stage reprogramming process.* BMC genomics, 2018. **19**(1): p. 1-17.
11. Okita, K., et al., *Generation of mouse-induced pluripotent stem cells with plasmid vectors.* Nature protocols, 2010. **5**(3): p. 418-428.
12. Zheng, G., et al., *ALKBH5 is a mammalian RNA demethylase that impacts RNA metabolism and mouse fertility.* Molecular cell, 2013. **49**(1): p. 18-29.
13. Aguilo, F., et al., *Coordination of m6A mRNA methylation and gene transcription by ZFP217 regulates pluripotency and reprogramming.* Cell stem cell, 2015. **17**(6): p. 689-704.
14. Batista, P.J., et al., *m6A RNA modification controls cell fate transition in mammalian embryonic stem cells.* Cell stem cell, 2014. **15**(6): p. 707-719.
15. Geula, S., et al., *m6A mRNA methylation facilitates resolution of naïve pluripotency toward differentiation.* Science, 2015. **347**(6225): p. 1002-1006.
16. Festuccia, N., et al., *Esrrb is a direct Nanog target gene that can substitute for Nanog function in pluripotent cells.* Cell stem cell, 2012. **11**(4): p. 477-490.
17. Loh, Y.-H., et al., *The Oct4 and Nanog transcription network regulates pluripotency in mouse embryonic stem cells.* Nature genetics, 2006. **38**(4): p. 431-440.
18. Boyer, L.A., et al., *Core transcriptional regulatory circuitry in human embryonic stem cells.* cell, 2005. **122**(6): p. 947-956.
19. Ma, Z., et al., *Sequence-specific regulator Prdm14 safeguards mouse ESCs from entering extraembryonic endoderm fates.* Nature structural & molecular biology, 2011. **18**(2): p. 120-127.

© 2022. Published by The Company of Biologists under the terms of the Creative Commons Attribution License (https://creativecommons.org/licenses/by/4.0/).

20. Yamaji, M., et al., *PRDM14 ensures naive pluripotency through dual regulation of signaling and epigenetic pathways in mouse embryonic stem cells.* Cell stem cell, 2013. **12**(3): p. 368-382.
21. Zhang, M., et al., *Esrrb complementation rescues development of Nanog-null germ cells.* Cell reports, 2018. **22**(2): p. 332-339.
22. Kim, S.-H., et al., *ERK1 phosphorylates Nanog to regulate protein stability and stem cell self-renewal.* Stem cell research, 2014. **13**(1): p. 1-11.
23. Marks, H., et al., *The transcriptional and epigenomic foundations of ground state pluripotency.* Cell, 2012. **149**(3): p. 590-604.

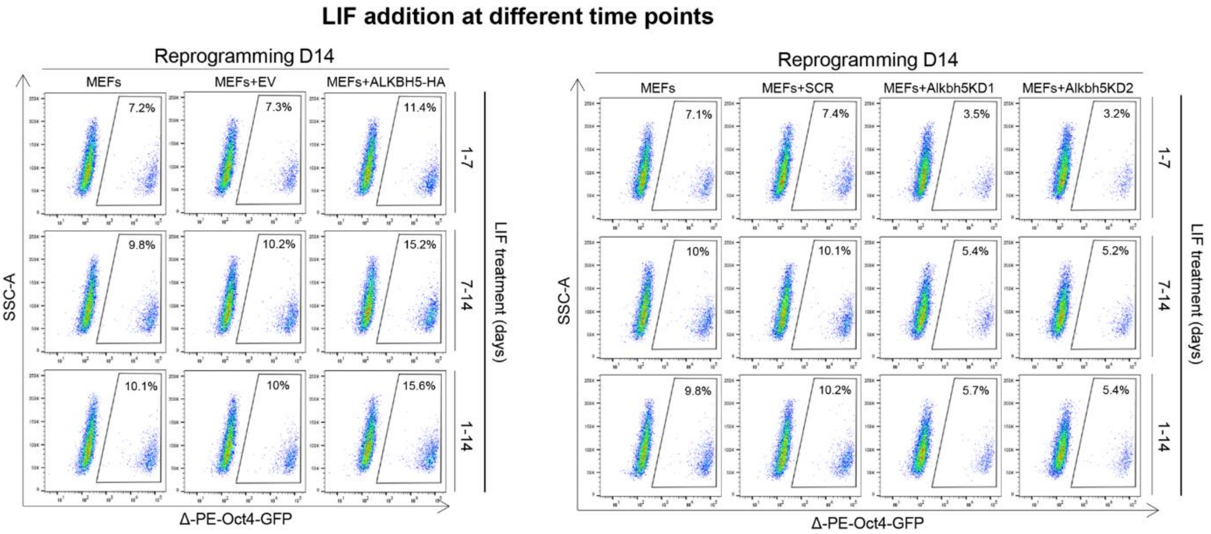

Our data revealed that, addition of LIF only at the early phase (day 1 to 7) of reprogramming has the lowest effect on reprogramming effiency. While addition of LIF at either late phase (day 7 to 14) or throught the whole reprogramming process (day 1 to 14) has a similar effect. All together , these data indicated that LIF is enhacning the reprogramming only at the late phase.

Original submission

First decision letter

MS ID#: JOCES/2022/259824

MS TITLE: ALKBH5 regulates somatic cell reprogramming in a phase specific manner

AUTHORS: Sherif Mahrous Khodeer, Arne Klungland, and John Arne Dahl
ARTICLE TYPE: Research Article

We have now reached a decision on the above manuscript.

To see the reviewers' reports and a copy of this decision letter, please go to: https://submit-jcs.biologists.org and click on the 'Manuscripts with Decisions' queue in the Author Area. (Corresponding author only has access to reviews.)

As you will see, the reviewers gave favourable reports but Reviewer 1 raised some minor points that will require amendments to your manuscript. I hope that you will be able to carry these out because I would like to be able to accept your paper.

We are aware that you may be experiencing disruption to the normal running of your lab that makes experimental revisions challenging. If it would be helpful, we encourage you to contact us to discuss your revision in greater detail. Please send us a point-by-point response indicating

© 2022. Published by The Company of Biologists under the terms of the Creative Commons Attribution License (https://creativecommons.org/licenses/by/4.0/).

where you are able to address concerns raised (either experimentally or by changes to the text) and where you will not be able to do so within the normal timeframe of a revision. We will then provide further guidance. Please also note that we are happy to extend revision timeframes as necessary.

Please ensure that you clearly highlight all changes made in the revised manuscript. Please avoid using 'Tracked changes' in Word files as these are lost in PDF conversion.

I should be grateful if you would also provide a point-by-point response detailing how you have dealt with the points raised by the reviewers in the 'Response to Reviewers' box. Please attend to all of the reviewers' comments. If you do not agree with any of their criticisms or suggestions please explain clearly why this is so.

Reviewer 1

Advance summary and potential significance to field

The authors investigated the effect of Alkbh5 depletion on cellular reprogramming and found that depletion of Alkbh5 during early stages of reprogramming lead to a reduction in Cyclin B1 and B2, and to G2/M arrest, therefore reducing the reprogramming efficiency. Depletion of Alkbh5 at the late stage of reprogramming does not affect the reprogramming efficiency. On the other hand, over-expression of Alkbh5 enhances reprogramming efficiency when done during late stages of reprogramming, probably by stabilizing Nanog expression.

The authors present rigorous and clean genetic study. I like the fact that they use multiple depletion methods, cell lines, reporters and methods of measurement to deliver a robust message, and I find this paper highly worthy of publication in your journal, and interesting for the research community. The paper is written in a clear way and is well explained. The conclusions are supported by the results.

Comments for the author

The authors investigated the effect of Alkbh5 depletion on cellular reprogramming and found that depletion of Alkbh5 during early stages of reprogramming lead to a reduction in Cyclin B1 and B2, and to G2/M arrest, therefore reducing the reprogramming efficiency. Depletion of Alkbh5 at the late stage of reprogramming does not affect the reprogramming efficiency. On the other hand, over-expression of Alkbh5 enhances reprogramming efficiency when done during late stages of reprogramming, probably by stabilizing Nanog expression.
The authors present rigorous and clean genetic study. I like the fact that they use multiple depletion methods, cell lines, reporters and methods of measurement to deliver a robust message, and I find this paper highly worthy of publication in your journal, and interesting for the research community. The paper is written in a clear way and is well explained. The conclusions are supported by the results.
Major comments: none Minor comments:
P8 L5: “Fig 1J” should be Fig 1G,H?
P8 L22: I would write “Next, we asked” and not “Then, we asked”
P9 L9: overexpression on WT ALKBH5-WT - on which day is the over expression?
(discussed later, but here too the information is required).
P11 L13: Should be “Fig3I-L”
P12 L16: Should be “as epithelial markers on day 6”
P15 L2: missing reference to Fig 4E
P15 L22: Should be “(Supplementary Fig 5B)”
P16 L14: Should be Fig 6A,B
Material and Methods: Please go over it again and fix spelling, typos, commas etc.
P34 L25 : Should be (J)
P36 L5: Should be Figure 3A
P44 L6: Should be Supplementary Fig 3A
In general: please emphasise in the result section that the reprogramming experiments were done as a single-cell-per-well, not only in the methods.

© 2022. Published by The Company of Biologists under the terms of the Creative Commons Attribution License (https://creativecommons.org/licenses/by/4.0/).

Fig 3F,H: There is a small increase in the transcript level of PDGFR8 but not in the protein, please discuss.

Reviewer 2

Advance summary and potential significance to field

The key findings of this paper are that the RNA m6A demethylase, ALKBH5 has different temporal effects on reprogramming of murine somatic cells to induced pluripotent stem cells. Using shRNA mediated knockdown or conditional knockout fibroblasts to deplete ALKBH5 they show that the depletion of ALKBH5 at the early stages of reprogramming (day 1-7) can reduce the generation of iPSCs. The overexpression of ALKBH5 at late stages of reprogramming (day 7-14) can increase the generation of AP+ colonies. The reduction sof reprogramming is due at least in part to alterations in cell cycle and reduced transition to an epithelial state.
The enhancement of reprogramming at later stages is through the stabilization of Nanog transcripts. Overall this study add an important piece of epitranscriptomic regulation to the mechanism of induced pluripotency.

Comments for the author

The authors have done a thorough, careful and logical job of addressing my prior concerns. I commend them for their efforts.

First revision

Author response to reviewers' comments

Reviewer 1 Advance summary and potential significance to field...

The authors investigated the effect of Alkbh5 depletion on cellular reprogramming, and found that depletion of Alkbh5 during early stages of reprogramming lead to a reduction in Cyclin B1 and B2, and to G2/M arrest, therefore reducing the reprogramming efficiency. Depletion of Alkbh5 at the late stage of reprogramming does not affect the reprogramming efficiency. On the other hand, over-expression of Alkbh5 enhances reprogramming efficiency when done during late stages of reprogramming, probably by stabilizing Nanog expression.

The authors present rigorous and clean genetic study. I like the fact that they use multiple depletion methods, cell lines, reporters and methods of measurement to deliver a robust message, and I find this paper highly worthy of publication in your journal, and interesting for the research community. The paper is written in a clear way and is well explained. The conclusions are supported by the results.

Reviewer 1 Comments for the Author...

The authors investigated the effect of Alkbh5 depletion on cellular reprogramming, and found that depletion of Alkbh5 during early stages of reprogramming lead to a reduction in Cyclin B1 and B2, and to G2/M arrest, therefore reducing the reprogramming efficiency. Depletion of Alkbh5 at the late stage of reprogramming does not affect the reprogramming efficiency. On the other hand, over-expression of Alkbh5 enhances reprogramming efficiency when done during late stages of reprogramming, probably by stabilizing Nanog expression.

The authors present rigorous and clean genetic study. I like the fact that they use multiple depletion methods, cell lines, reporters and methods of measurement to deliver a robust message, and I find this paper highly worthy of publication in your journal, and interesting for the research community. The paper is written in a clear way and is well explained. The conclusions are supported by the results.

© 2022. Published by The Company of Biologists under the terms of the Creative Commons Attribution License (https://creativecommons.org/licenses/by/4.0/).

✓ We thank reviewer (1) for his positive comment and we appreciate his positive feedback.

Major comments: none.

Minor comments:

- P8 L5: "Fig 1J" should be Fig 1G,H?

We have now corrected this in the revised manuscript and highlighted in P7 L13.

- P8 L22: I would write "Next, we asked" and not "Then, we asked"

We have now edited this in the revised manuscript and highlighted in P8 L5.

- P9 L9: overexpression on WT ALKBH5-WT - on which day is the over expression?

(discussed later, but here too the information is required).

We have now added the missing information "on day 1 of reprogramming" in the revised manuscript and highlighted in P8 Line 22 and 23.

- P11 L13: Should be "Fig3I-L"

We have now corrected this in the revised manuscript and highlighted in P10 L22.

- P12 L16: Should be "as epithelial markers on day 6"

We have now corrected this to be "thymocyte differentiation antigen-1 (Thy-1) as a mesenchymal marker on day 6" in the revised manuscript and highlighted in P11 L25 and P12 L1.

- P15 L2: missing reference to Fig 4E

We have now corrected this in the revised manuscript and highlighted in P14 L13.

- P15 L22: Should be "(Supplementary Fig 5B)"

We have now corrected this in the revised manuscript and highlighted in P15 L9.

- P16 L14: Should be Fig 6A,B

We have now corrected this in the revised manuscript and highlighted in P16 L1.

- Material and Methods: Please go over it again and fix spelling, typos, commas etc.

We have now gone through the material and methods section and fixed this in the revised manuscript and highlighted.

- P34 L25 : Should be (J)

We have now corrected this in the revised manuscript and highlighted in P37 L25.

- P36 L5: Should be Figure 3A

We have now corrected this in the revised manuscript and highlighted in P39 L5.

- P44 L6: Should be Supplementary Fig 3A

We have now corrected this in the revised manuscript and highlighted in supplementary figures P11 L6.

© 2022. Published by The Company of Biologists under the terms of the Creative Commons Attribution License (https://creativecommons.org/licenses/by/4.0/).

- In general: please emphasise in the result section that the reprogramming experiments were done as a single-cell-per-well, not only in the methods.

We thank the reviewer for his point. However, we did not mention in any part of our methods that we did the experiments as a single cell per well. All reprogramming experiments have been done by plating indicated number of cells either in 24, 12, or 6 well plates before reprogramming or after reprogramming for alkaline phosphatase (AP) staining as explained on methods part.

- Fig 3F,H: There is a small increase in the transcript level of PDGFR8 but not in the protein, please discuss.

We have added the following statement “It is noteworthy that the discrepancy between PDGFRß RNA and protein levels might due to posttranscriptional regulation.” in the revised manuscript and highlighted in P12 L11-13.

Reviewer 2 Advance Summary and Potential Significance to Field...

The key findings of this paper are that the RNA m6A demethylase, ALKBH5 has different temporal effects on reprogramming of murine somatic cells to induced pluripotent stem cells. Using shRNA mediated knockdown or conditional knockout fibroblasts to deplete ALKBH5 they show that the depletion of ALKBH5 at the early stages of reprogramming (day 1-7) can reduce the generation of iPSCs. The overexpression of ALKBH5 at late stages of reprogramming (day 7-14) can increase the generation of AP+ colonies. The reduction of reprogramming is due at least in part to alterations in cell cycle and reduced transition to an epithelial state.

The enhancement of reprogramming at later stages is through the stabilization of Nanog transcripts. Overall, this study adds an important piece of epitranscriptomic regulation to the mechanism of induced pluripotency.

Reviewer 2 Comments for the Author...

The authors have done a thorough, careful, and logical job of addressing my prior concerns. I commend them for their efforts.

- ✓ We thank reviewer 2 for his positive comment, and we appreciate his time spent on our manuscript

---

Second decision letter

MS ID#: JOCES/2022/259824

MS TITLE: ALKBH5 regulates somatic cell reprogramming in a phase specific manner

AUTHORS: Sherif Mahrous Khodeer, Arne Klungland, and John Arne Dahl
ARTICLE TYPE: Research Article

Thank you for sending your manuscript to Journal of Cell Science through Review Commons.

I am happy to tell you that your manuscript has been accepted for publication in Journal of Cell Science, pending standard ethics checks.

© 2022. Published by The Company of Biologists under the terms of the Creative Commons Attribution License (https://creativecommons.org/licenses/by/4.0/).
